# Designer Moiré Quantum Materials Enabled by Freestanding Oxide Membranes in Twisted and Hybrid Bilayers

**DOI:** 10.1002/advs.77727

**Published:** 2026-09-11

**Authors:** Puneet Kaur, Rahul, Jan‐Chi Yang

**Affiliations:** ^1^ Department of Physics National Cheng Kung University Tainan Taiwan; ^2^ Program on Key Materials Academy of Innovative Semiconductor and Sustainable Manufacturing (AISSM) National Cheng Kung University Tainan Taiwan; ^3^ Center for Quantum Frontiers of Research & Technology (QFort) National Cheng Kung University Tainan Taiwan

**Keywords:** flat bands, freestanding oxide membrane, hybrid oxide‐2D moiré, moiré superlattices, oxide twistronics, quantum materials

## Abstract

With the increasing demand for emergent functionalities in next‐generation electronic devices, materials research has shifted from conventional materials discovery toward structural reconstruction and interface engineering. In this regime, the discovery of moiré superlattices has established twistronics as a powerful platform for generating emergent phenomena through twist‐induced modulations of atomic registry and interfacial coupling. While most twist‐engineered systems have been confined to van der Waals materials, extending them to strongly correlated complex oxides represents a challenging yet promising frontier. Recent developments in freestanding oxide membranes have enabled this transition by eliminating substrate‐imposed epitaxial constraints and allowing deterministic stacking and twisting of crystalline oxide thin films. Experiments have established structural moiré superlattices, polar textures, and registry‐dependent charge disproportionation. In contrast, flat bands, charge ordering, and tunable magnetic interactions remain primarily theoretical predictions requiring direct experimental verification. Hybrid oxide‐two‐dimensional (2D) heterostructures further combine correlated oxides degree of freedom with the tunable electronic and optical properties of 2D materials, opening new opportunities for unconventional moiré physics. In this review, we summarize the recent advances in oxide twistronics, spanning freestanding oxide membranes, twisted oxide bilayers, and hybrid oxide‐2D moiré heterostructures, while highlighting future direction for engineering emergent quantum phases and next‐generation multifunctional device architectures.

## Graphical abstract

Freestanding oxide membranes transform twistronics by enabling deterministic stacking, strain release, and integration with two‐dimensional materials. A unified framework links local atomic registry to structural reconstruction, polar textures, charge modulation, flat bands, and magnetic responses, while identifying the fabrication and characterization challenges that govern the emergence of scalable oxide moiré quantum platforms.

## 1 Introduction—From Twistronics to Oxide Moiré Systems

Herbert Kroemer's famous statement, “The interface is the device,” has evolved from a guiding principle in semiconductor physics into a central paradigm for contemporary quantum materials research [1]. Far from being passive boundaries, interfaces constitute electronically and structurally distinct environments where lattice geometry, charge transfer, orbital hybridization, and symmetry breaking become intimately coupled across atomic length scales. These interfacial interactions can fundamentally reconstruct the local energy landscape, giving rise to emergent phenomena that are inaccessible in the parent bulk constituents alone. As a result, the deliberate engineering of interfaces has become a powerful strategy for creating and controlling novel quantum states, enabling unprecedented opportunities for the discovery of correlated, topological, ferroic, and low‐dimensional functionalities. Building on this broader context of interface‐driven phenomena, oxide heterointerfaces can host electronic and magnetic states that are absent in their individual bulk constituents. A seminal example is the LaAlO_3_/SrTiO_3_ heterointerface, where Ohtomo and Hwang reported a high‐mobility interfacial electron gas [2]. Subsequent studies demonstrated two‐dimensional superconductivity and reported magnetic transport signatures at this interface, while spatially resolved magnetic measurements later revealed inhomogeneous ferromagnetic regions [3, 4, 5]. These discoveries established oxide interfaces as an important platform for realizing emergent conductivity, superconductivity, and magnetism. In this light, oxide twistronics can be viewed as a natural extension of conventional interface engineering, introducing relative rotation and the resulting spatial modulation of atomic registry as additional structural control parameters [6].

Complex oxides host a broad range of electronic, magnetic, and ferroic functionalities, including superconductivity, multiferroicity, ferromagnetism, piezoelectricity, colossal magnetoresistance, and metal‐insulator transitions [7, 8]. This intrinsic multifunctionality, together with the diversity of interfacial phenomena described above, originates from the strong coupling among lattice, charge, spin, and orbital degrees of freedom in complex oxides [3, 8, 9]. Consequently, their electronic, magnetic, and ferroic ground states can be highly sensitive to variations in bond lengths, bond angles, crystal symmetry, carrier concentration, and defect chemistry. Epitaxial thin‐film synthesis is therefore not merely a route for preparing crystalline oxides, but also a means of modifying their free energy landscape. For example, coherent biaxial strain can stabilize functional states that are absent in the corresponding unstrained bulk material and can modify oxygen‐octahedral rotation patterns by changing the lattice geometry [10, 11]. Film thickness and heterostructure periodicity provide additional control by tuning the degree of dimensional confinement and the spatial repetition of the interfaces, thereby influencing the quantum‐confinement effects, interlayer coupling, and emergence of collective electronic phases [12]. Careful control of cation composition and oxygen stoichiometry is equally important, because relatively small deviations can change carrier density, transition‐metal valence, crystal structure, electrical transport, and magnetic order [13, 14]. The choice of surface termination and layer sequence can further alter the atomic stacking sequence, local coordination, and electrostatic boundary conditions across an oxide heterointerface [15]. Modern epitaxial techniques, particularly pulsed‐laser deposition and molecular‐beam epitaxy, thus provide multiple routes for manipulating oxide phases through control of structural, chemical, and interfacial boundary conditions [9].

However, the same substrate‐imposed boundary conditions that make epitaxy a powerful means of controlling oxide properties also restrict the accessible material combinations and structural configurations. Epitaxial films remain coupled to their growth substrates through mechanical clamping, lattice compatibility, and interfacial structural continuity. These constraints can limit strain relaxation and make it difficult to combine crystalline materials that require mutually incompatible substrates, orientations, or growth conditions.

Freestanding (FS) oxide membranes provide a distinctive solution by decoupling the substrate required for epitaxial synthesis from the substrate or material used for subsequent integration. In this approach, the functional oxide is first grown under conditions that establish high crystalline quality and is then released from the original substrate while retaining its single‐crystalline structure [16, 17]. Removal of the original epitaxial clamping allows the mechanical and structural boundary conditions to be relaxed or redefined through transfer, bending, strain application, or stacking [17, 18]. The released membrane can also be integrated with substrates and materials that would not withstand, or would not be crystallographically compatible with, its original high‐temperature growth process [19]. Most importantly for oxide twistronics, freestanding membranes can be translated, rotated, and restacked after growth, enabling interfacial orientations and twist angles that are not dictated by conventional epitaxial relationships [20, 21]. This combination of epitaxial crystallinity and post‐growth structural reconfigurability is the defining advantage of freestanding oxide membranes and establishes them as an enabling platform for twisted oxide bilayers and hybrid moiré heterostructures.

Beyond enabling post‐growth integration, freestanding oxide membranes provide several structural degrees of freedom relevant to oxide twistronics. Release from substrate clamping permits strain accommodation and mechanical deformation, while membrane thickness, lattice configuration, and interfacial geometry can be independently controlled. Transfer and deterministic restacking further enable integration with dissimilar oxides and two‐dimensional materials, the formation of twist‐defined moiré superlattices, and the reconstruction of ferroic order parameters. These interconnected capabilities are summarized schematically in Figure 1.

**FIGURE 1:**
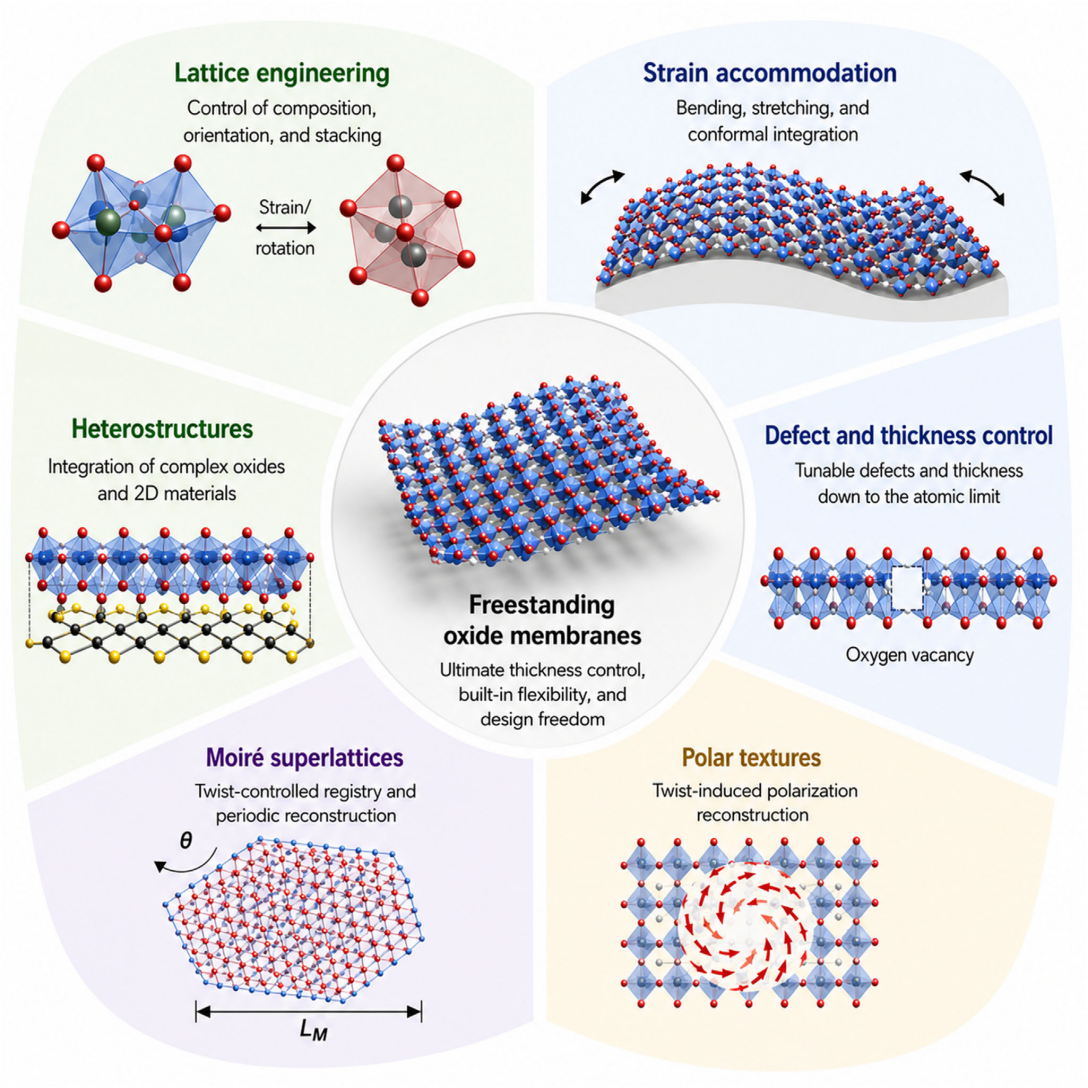
Freestanding oxide membranes as an enabling platform for twisted oxide and hybrid moiré systems. Removal of substrate clamping enables mechanical compliance, strain accommodation, and control over lattice configuration and membrane thickness. Transfer and deterministic restacking permit integration with dissimilar oxides and two‐dimensional materials, while relative rotation generates spatially varying atomic registry and moiré superlattices. Structural relaxation, reduced dimensionality, and defect chemistry provide additional parameters for modifying thin film properties. In twisted oxide bilayers, twist‐induced structural reconstruction can couple to polarization and generate nonuniform polar textures. The illustrations are conceptual representations of the principal structural and functional degrees of freedom discussed in this review.

FS oxide membranes are commonly produced using a sacrificial‐layer‐assisted lift‐off process, in which a selectively removable intermediate layer is inserted between the functional oxide film and its growth substrate. Dissolution or etching of this sacrificial layer releases the epitaxial film, which can subsequently be transferred onto a broad range of target substrates [22]. Because the high‐temperature epitaxial growth and the final materials integration are performed in separate steps, crystalline oxide membranes can be incorporated onto temperature‐sensitive, flexible, or otherwise crystallographically incompatible platforms [23, 24]. After transfer, adhesion to the target substrate is typically mediated by short‐range interfacial interactions, including van der Waals forces, although the resulting contact quality depends strongly on surface roughness, cleanliness, trapped contaminants, and transfer conditions [25, 26]. When sufficiently uniform and mechanically stable, transferred membranes can undergo subsequent device‐processing steps, including lithographic patterning, electrode fabrication, and etching. This growth‐and‐transfer strategy therefore offers a potential route for heterogeneous integration of crystalline functional oxides with silicon and complementary metal‐oxide‐semiconductor (CMOS)‐compatible platforms, contributing to emerging “More‐than‐Moore” device architectures [27, 28, 29, 30, 31].

More importantly for oxide twistronics, release from substrate clamping permits strain relaxation, symmetry lowering, and bond redistribution while retaining the strong coupling among structural, electronic, magnetic, and ferroic degrees of freedom. These changes can stabilize phases and textures that are inaccessible under conventional epitaxial constraints [17, 32, 33, 34, 35, 36, 37, 38, 39, 40]. The deterministic stacking and rotation of FS oxide membranes introduce further structural degrees of freedom, which enable the formation of twisted bilayer oxides and hybrid heterostructures, where periodic modulation of atomic registry gives rise to moiré superlattices. In contrast to the conventional van der Waals system, where weak interlayer coupling governs the moiré effects [41, 42], oxide moiré systems exhibit strong correlations between lattice reconstruction and correlated electronic behavior, leading to phenomena such as polar textures, charge modulation, flat‐band formation, and twist‐dependent interfacial coupling [20, 32, 43, 44, 45]. Furthermore, the integration of FS oxide membranes with two‐dimensional materials enables the realization of hybrid oxide‐two‐dimensional (2D) moiré systems, where interfacial coupling is governed by the combination of lattice mismatch, electrostatic interactions, and interfacial dipoles, extending the concept of twistronics beyond conventional van der Waals systems. These twisted oxide and hybrid oxide‐2D platforms provide new opportunities for engineering emergent quantum phenomena, including exciton minibands, charge transfer dynamics, and spin‐dependent interactions, positioning them as a versatile framework for designing next‐generation quantum materials and multifunctional device architectures beyond conventional epitaxy and van der Waals systems.

Several recent reviews have provided valuable perspectives on the fabrication, release, transfer, mechanical response, and heterogeneous integration of freestanding oxide membranes, together with the broader opportunities offered by stacked oxide architectures [46, 47, 48, 49]. Building on these important contributions, the present review adopts a complementary twistronics‐centered perspective, focusing on recent advances in twist‐defined oxide bilayers and hybrid oxide‐2D moiré systems. Particular emphasis is placed on local atomic registry, finite‐thickness interfacial reconstruction, polar textures, charge disproportionation, flat‐band and magnetic states, hybrid moiré excitonic states, and the experimental challenges associated with probing buried interfaces. This scope connects the technological development of freestanding membranes with the rapidly emerging physics of moiré‐engineered oxide quantum materials. In this review, we examine freestanding oxide membranes as a reconfigurable platform to engineer twisted oxide bilayers and their hybrid moiré superlattices. The possibility of eliminating the epitaxial constraints in freestanding thin films redefines hetero‐ and homo‐structure designs, facilitating precise control over stacking configuration, twisting, and interfacial coupling. These structural reconfigurations unlock a new structural degree of freedom, which gives rise to a moiré potential and numerous emergent phenomena governed by local atomic registry modulations. In contrast to the conventional moiré superlattices based on van der Waals materials, these oxide‐based architectures host strongly correlated electronic states, emerging from the intricate coupling between lattice distortions, coupled charge, spin, and orbital degrees of freedom. Thus, this results in intrinsically complex and strongly coupled material systems that possess a substantial challenges for experimental and theoretical investigation. To provide a comprehensive perspective on this rapidly evolving field, Section 2 delves into the historical evolution of FS techniques, tracing their development from early methodologies to current state‐of‐the‐art approaches. Section 3 introduces the concept of geometry‐driven interfacial physics in oxide bilayers, establishing the fundamental framework for realizing twist‐ and stacking‐dependent phenomena. Section 4 focuses on twisted oxide bilayers and discusses the emergence of correlated electronic states, polar vortex structures, and interfacial magnetic interactions enabled by moiré engineering. Section 5 extends this paradigm to the utilization of FS oxide thin films to design unconventional hybrid moiré systems comprising oxides and 2D transition metal dichalcogenides (TMDs). The stacking and twisting of these dissimilar materials introduce additional pathways to tune electronic, ferroic, and optical responses. Finally, Section 6 addresses the key experimental and theoretical challenges currently limiting progress in this field and outlines future opportunities for engineering designer moiré materials with tailored functionalities. Throughout this review, experimentally measured phenomena are distinguished from theoretical predictions. Structural moiré superlattices, selected polar textures, registry‐dependent charge disproportionation, and hybrid moiré excitonic states are discussed as experimentally established results. By contrast, oxide moiré flat bands, orbital ordering, twist‐modulated magnetic states, and interaction‐driven correlated phases are identified as predictions that remain to be directly verified. Overall, this review highlights the emergence of geometry‐driven materials design as a new paradigm in condensed matter and quantum materials research, where stacking and twisting serve as powerful tools for creating functionalities beyond those attainable in conventional epitaxial heterostructures.

## 2 Freestanding Oxide Membranes as an Enabling Platform

Freestanding crystalline films can be produced through optical, mechanical, fracture‐assisted, or chemically selective release processes. The applicability of each approach is determined by the bonding character of the parent material, optical properties of the film‐substrate stack, fracture mechanics, availability of a selectively removable interlayer, and the required membrane dimensions. Laser lift‐off was developed primarily for III‐nitride films on optically transparent substrates [50, 51, 52, 53, 54], whereas conventional mechanical exfoliation is most effective for intrinsically layered materials containing weak van der Waals cleavage planes. Controlled spalling relies on stress‐driven subsurface fracture and has been applied mainly to brittle semiconductor wafers. In contrast, sacrificial‐layer‐assisted chemical lift‐off has become the most widely developed route for ultrathin epitaxial complex‐oxide membranes because it enables selective release while retaining the thickness, orientation, and crystallinity established during epitaxial growth. Table 1 summarizes the applicable material classes, release mechanisms, advantages, limitations, scalability, and typical film quality associated with these approaches.

**TABLE 1:** Comparative overview of laser lift‐off, mechanical exfoliation, controlled spalling, and sacrificial‐layer‐assisted chemical lift‐off for producing freestanding crystalline films, summarizing their release mechanisms, applicable material classes, advantages, limitations, scalability, typical film quality, and representative foundational studies.

Release method	Release mechanism	Principal material classes	Advantages	Limitations	Scalability and typical film quality	Refs.
Laser Lift Off	A pulsed laser is transmitted through an optically transparent substrate and absorbed at the film‐substrate interface or in a designated release layer, causing localized decomposition or ablation.	Primarily GaN and related III‐nitrides on sapphire; selected semiconductor and oxide stacks satisfying the required optical and thermal conditions.	Rapid and dry; no prolonged lateral etching; compatible with wafer‐level processing.	Requires an appropriate optical‐absorption contrast and usually a transparent carrier substrate; localized heating can produce interfacial roughening, decomposition products, residual stress, or structural damage.	Wafer‐scale release has been demonstrated for III‐nitrides. Single‐crystalline films can be retained, although the released surface may require cleaning or post‐processing.	[55, 56]
Mechanical Exfoliation	Directly applicable to intrinsically layered van der Waals crystals; applicable to non‐layered epitaxial semiconductors and complex oxides only when a mechanically weak interface, such as graphene, is intentionally introduced	Naturally layered van der Waals crystals; semiconductors and complex oxides grown using graphene‐assisted or related weak‐interface epitaxy.	Etchant‐free and potentially rapid; can preserve the growth substrate; avoids chemical exposure of the membrane.	Direct exfoliation is generally unsuitable for epitaxial complex oxides because they lack intrinsic van der Waals gaps; 2Dlayer‐assisted release requires a continuous, growth‐compatible release layer.	High‐quality single‐crystalline membranes are possible, but yield and uniformity depend on release‐layer integrity, defects, pinholes, interfacial adhesion, and film stress.	[57, 58]
Controlled Spalling	A tensile stressor layer drives a subsurface crack parallel to the surface, releasing a film whose thickness is controlled by the stress state and fracture mechanics.	Brittle Si, Ge, III‐V semiconductors, and GaN; comparatively limited application to ultrathin epitaxial complex oxides.	Room‐temperature process; no sacrificial layer or wet etchant; substrate reuse and wafer‐scale release are possible.	Crack depth and uniformity depend sensitively on stressor thickness, residual stress, fracture toughness, and crystallographic orientation; surface roughness, bowing, unintended cracking, and limited nanometer‐scale thickness control may occur.	Wafer‐scale release has been demonstrated for several semiconductor systems. Device layers can retain functionality, although the fracture surface may require further processing.	[59, 60]
Chemical or sacrificial layer lift‐off	The substrate or an epitaxial sacrificial layer is selectively dissolved while the functional film is supported by a polymer, stamp, or target substrate.	III‐V semiconductors and epitaxial complex oxides for which a chemically selective substrate or sacrificial layer can be identified.	Film thickness and crystallinity are inherited from epitaxial growth; compatible with ultrathin membranes, multilayers, and heterostructures; enables deterministic transfer to dissimilar substrates.	Requires high chemical selectivity and compatibility among the film, sacrificial layer, and etchant; lateral etching may be slow. Polymer residue, cracks, wrinkles, chemical modification, and oxygen‐stoichiometry changes can arise during release and transfer.	Millimeter‐ to centimeter‐scale oxide membranes have been demonstrated. Single‐crystalline films down to the unit‐cell limit can be retained, although large‐area defect‐free transfer remains challenging.	[17, 61]

The comparison indicates that laser lift‐off, conventional mechanical exfoliation, and controlled spalling were initially developed predominantly for III‐nitrides, layered crystals, and brittle semiconductor wafers, respectively. Their extension to complex oxides therefore requires specific optical, interfacial, or fracture conditions. In particular, conventional epitaxial complex oxides generally lack weak van der Waals gaps, and their three‐dimensionally connected ionic‐covalent lattices form strongly bonded interfaces with the growth substrates. Consequently, these films cannot usually be released through direct exfoliation without an intentionally engineered weak interface, such as a two‐dimensional release layer. The absence of an intrinsic cleavage plane is therefore the fundamental limitation, whereas reproducibility, membrane dimensions, and film integrity represent subsequent practical challenges. By contrast, selective chemical lift‐off using epitaxially compatible sacrificial layers has enabled the release of ultrathin single‐crystalline oxide films and heterostructures while retaining their epitaxially defined thickness and orientation. Its effectiveness nevertheless depends on sacrificial‐layer compatibility, etching selectivity and kinetics, membrane handling, and preservation of surface chemistry and oxygen stoichiometry.

In sacrificial‐layer‐assisted chemical lift‐off, an epitaxially grown release layer located between the substrate and the functional oxide film is selectively removed using an etchant that is chemically compatible with the functional membrane, as illustrated in Figure 2a. Following the deposition, a chosen selective etchant, which is chemically inert to the functional film, etches the sacrificial layer, thus releasing the high‐quality FS thin film. The composition of the sacrificial layer, type and concentration of etchant, and duration of etching are some of the vital parameters that can greatly affect the quality of the etched film. For fabricating large‐area crack‐free FS thin films, usually a protective polymer such as Polydimethylsiloxane (PDMS) or Polymethylmethacrylate (PMMA) is used, as demonstrated in Figure 2b,c [62]. Although polymer supports improve membrane integrity during release, the lift‐off and transfer processes can introduce several structural and chemical artifacts. Incomplete removal of PMMA or PDMS may leave polymer‐derived residues, while trapped water, air, or adsorbates can form bubbles or interfacial pockets. Capillary forces during wet release and drying, relaxation of residual epitaxial strain, and nonuniform adhesion to the target substrate can generate wrinkles, delamination, and cracks. Surface roughness and step‐terrace mismatch may additionally prevent conformal contact and create local interfacial gaps. For chemically sensitive oxides, prolonged exposure to water or etchants may also modify surface termination or defect chemistry, including oxygen stoichiometry. These effects can alter local strain, interfacial separation, charge transfer, transport, and ferroic response and must therefore be distinguished from intrinsic moiré‐induced spatial variations [63, 64, 65].

**FIGURE 2:**
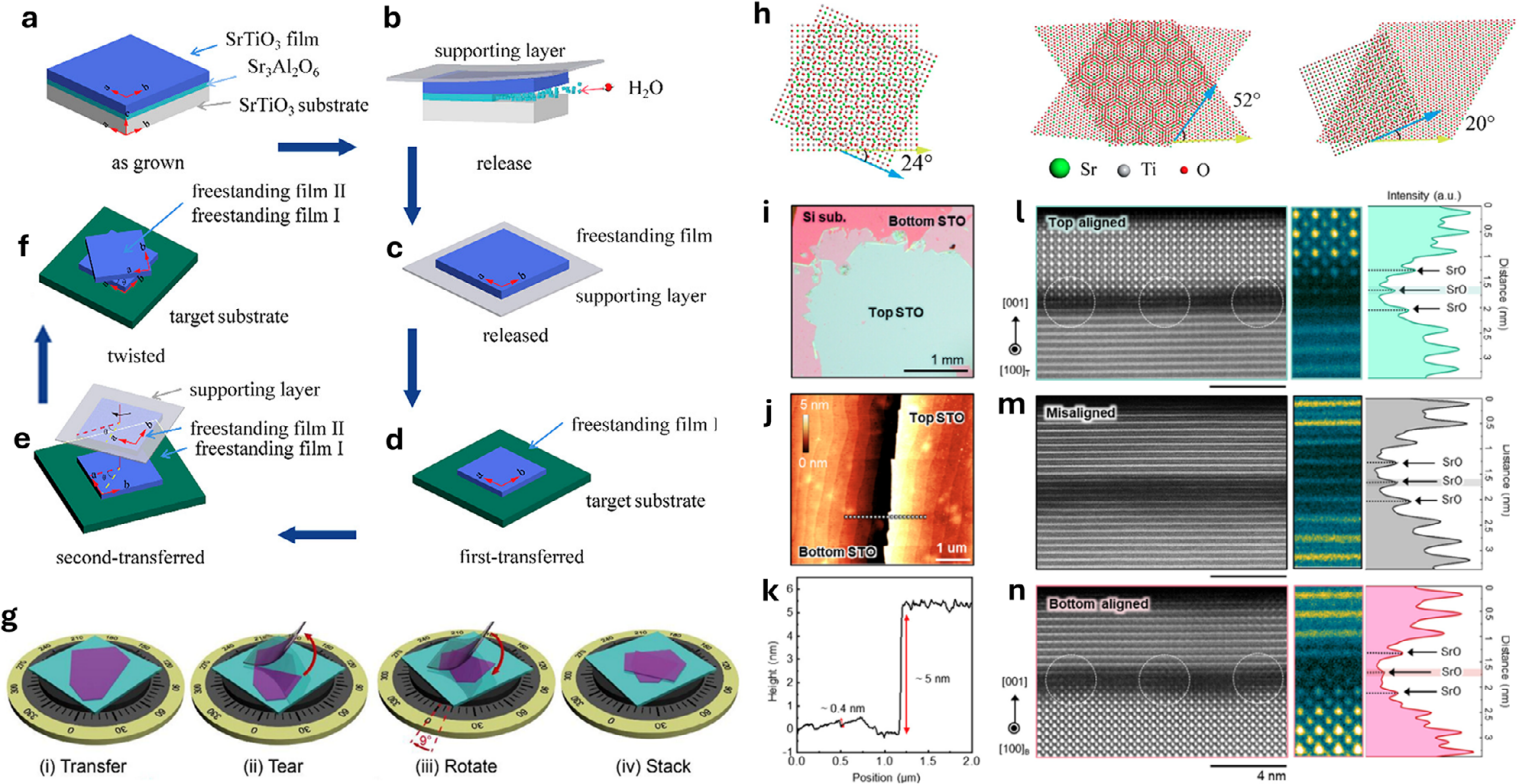
Freestanding oxide membrane fabrication and twist assembly. (a) Epitaxial growth of the STO film on a STO single‐crystal substrate with SAO as a sacrificial layer. Reproduced with permission [20]. Copyright 2022, American Chemical Society. (b) Release of the freestanding membrane using a polymer‐assisted lift‐off process via selective etching of the sacrificial layer. Reproduced with permission [20]. Copyright 2022, American Chemical Society. (c) FS thin film on a PDMS supporting layer. Reproduced with permission [20]. Copyright 2022, American Chemical Society. (d) Transfer of the freestanding oxide film onto a Si substrate, and the supporting layer is removed by the thermal release method. Reproduced with permission [20]. Copyright 2022, American Chemical Society. (e) Deterministic stacking and alignment of a second freestanding membranes using a transfer platform to control the twist angle. Reproduced with permission [20]. Copyright 2022, American Chemical Society. (f) Schematic of the resulting twisted bilayer structure, illustrating the relative lattice orientation used to define the twist angle. Reproduced with permission [20]. Copyright 2022, American Chemical Society. (g) Schematic of the tear and stack method. Reproduced with permission [66]. Copyright 2025, John Wiley and Sons. (h) Schematic of simulated moiré pattern in STO(100)/STO(100), STO(111)/STO(111), and STO(111)/STO(100) bilayers twisted at 24°, 52°, and 20° respectively. Reproduced with permission [67]. Copyright 2022, American Chemical Society. (i) Optical image of twisted STO bilayers. Reproduced with permission [67]. Copyright 2025, American Chemical Society. (j, k) AFM topography and corresponding height profile across the boundary between top and bottom STO membranes, confirming successful stacking and interface formation. Reproduced with permission [67]. Copyright 2025, American Chemical Society. (l–n) Atomic‐scale HAADF‐STEM images and corresponding line profiles of the bilayer interface for different alignment configurations, top‐aligned, misaligned, and bottom‐aligned, revealing the variation in interfacial structure and zone‐axis tilt, with the relative tilt between top and bottom layers corresponding to the twist angle. Reproduced with permission [67]. Copyright 2025, American Chemical Society.

Recently, complex oxide‐based sacrificial layers have gained attention, particularly due to their compatibility with the existing epitaxial growth techniques. The selection of the oxide sacrificial layer is critical, as it provides a coherent growth template while also allowing selective removal during the etching process. It is primarily important to maintain the lattice integrity during the deposition and lift‐off procedure to achieve high‐quality single‐crystal FS thin films. A variety of complex oxides, including SrRuO_3_ (SRO), Sr_3_Al_2_O_6_ (SAO), La_0.7_Sr_0.3_MnO_3_ (LSMO)_,_ Ca_3_Al_2_O_6_ (CAO), YBa_2_Cu_3_O_7‐x_ (YBCO), Ba_3_Al_2_O_6_ (BAO), (Ca,Sr)_3_Al_2_O_6_ (CSAO), have been employed as sacrificial layers [16, 22, 68, 69]. An early demonstrations were reported by Gan et al. (1988), where a selectively etched SrTiO_3_ (STO) substrate was immersed in an acid solution to fabricate an FS‐SRO thin film [70]. Building this concept further, selective removal of LSMO was commonly employed to fabricate freestanding Pb(Zr_0.2_Ti_0.8_)O_3_ (PZT) thin films, and it was further extended to obtain complex oxide superlattices and multilayer heterostructures [69]. Such FS thin films have been successfully integrated into device architecture, for example, as a dielectric layer in silicon‐based transistors, resulting in enhanced device performance.

Despite these advancements, the utilization of acid‐based etchants can limit the compatibility and comprehensive usage of the chemical lift‐off procedure. To broaden the applicability of the chemical lift‐off process and to ensure a damage‐free lift‐off process, hygroscopic and water‐soluble SAO has been developed as a sacrificial layer [16, 71]. The lattice tunability of SAO‐based systems further provides a tunable window for strain engineering across a wide range of perovskite oxides [68]. However, the slow etching rates often limit the applicability of SAO as a sacrificial layer, as sometimes the etching time extends to several days. In contrast, YBCO emerged as a highly selective sacrificial layer with a fast‐etching rate, although it still relies on acid‐based etching solutions [22].

With the development of diverse sacrificial layer chemistry, these FS membranes facilitate the direct fabrication route for twisted architectures through twist‐and‐stack mechanisms. As demonstrated in Figure 2d–f, it is feasible to stack a second FS layer onto an existing FS layer with deterministic control of interlayer twist angle. This controlled rotational misalignment enables the formation of twisted bilayers with a defined twist angle and introduces spatial variation in atomic registry, which leads to the formation of moiré superlattices (Figure 2f) [20]. Once released from their growth substrates, freestanding oxide membranes can be assembled into twisted bilayers using either sequential transfer or tear‐and‐stack approaches. In the conventional sequential‐transfer method illustrated in Figure 2d–f, two membranes are released and handled independently. The first membrane is transferred onto the target substrate, after which the second membrane is rotationally aligned and placed on top. This approach provides broad design flexibility because the constituent layers can be selected independently in terms of composition, thickness, crystallographic orientation, strain state, and growth conditions. However, separate release, support, alignment, and transfer of the two membranes can introduce cumulative uncertainties in the final twist angle and increase the possibility of polymer residue, trapped adsorbates, wrinkles, or local interfacial gaps.

More recently, Zhang et al. introduced a tear‐and‐stack workflow for freestanding complex oxides as shown in Figure 2g [66]. In the reported STO implementation, a freestanding parent membrane was first transferred onto a Si substrate. A portion of the membrane was then torn and picked up using a polycarbonate‐supported stamp, rotated by the prescribed angle, and restacked onto the remaining part of the same membrane. Because the overlapping layers originated from a common parent film, variations in composition, thickness, crystallographic orientation, and growth history were intrinsically minimized. The method provided a reported angular‐control accuracy of approximately 0.1°. Following assembly, oxygen flash annealing was used to promote interfacial recrystallization and eliminate the amorphous nanogap initially present between the transferred oxide layers, resulting in an atomically sharp twisted STO interface [66].

Sequential transfer and tear‐and‐stack therefore provide complementary forms of control. Sequential transfer permits independent selection of the two constituent membranes and is directly applicable to compositionally or structurally dissimilar bilayers. By contrast, the reported same‐parent tear‐and‐stack configuration improves structural equivalence between the overlapping regions and reduces uncertainties arising from independently prepared membranes. The tear‐and‐stack concept is not intrinsically restricted to homobilayers, however, its extension to dissimilar oxide layers would require adaptation of the demonstrated same‐parent pickup and restacking workflow. In both approaches, the final interface quality remains sensitive to membrane flatness, surface cleanliness, transfer‐induced deformation, trapped contaminants, and post‐stacking treatment.

Irrespective of the assembly route, the symmetry, periodicity, and structural complexity of the resulting moiré pattern are governed by the projected surface lattices, lattice mismatch, relative twist angle, and interfacial relaxation. Identical lattice symmetry is not a prerequisite for moiré formation, rotational misalignment and lattice mismatch between dissimilar lattices can also generate moiré structures. However, symmetry‐compatible lattices generally favor well‐defined and regularly periodic moiré superlattices. As illustrated in Figure 2h, twisted STO(100)/STO(100) and STO(111)/STO(111) bilayers produce comparatively regular moiré patterns that reflect the square and hexagonal symmetries of their respective surface lattices. In contrast, stacking symmetry‐inequivalent STO(100) and STO(111) membranes generate a more complex moiré configuration arising from the superposition of square and hexagonal lattices [67].

The recent advances in twisted bilayer assembly demonstrate that it is possible to achieve atomically sharp and clean interfaces, even with the film thickness of a few nm (Figure 2i–k). This indicates that the wet etching transfer route is highly robust and compatible with ultrathin oxide membranes, specifically when the interfacial effects are increasingly dominant. High‐resolution high‐angle annular dark‐field scanning transmission electron microscopy (HAADF‐STEM) clearly resolves the top and bottom crystalline layers alongside the interface region, providing direct structural evidence for these STO twisted bilayers. By selectively aligning the electron beam along the zone axis of the top or bottom layer (Figure 2l,n), a relative lattice tilt of ∼10.4° is observed, corresponding to the twist angle between the two layers. In contrast, imaging along an intermediate zone axis (Figure 2m) reveals a reduced tilt of ∼5°, consistent with a projection between the two orientations. Essentially, the periodic modulations at the interface indicate the formation of moiré patterns arising from rotational misalignment between two STO films [67].

## 3 Geometry‐Driven Interfacial Physics in Oxide Bilayers

In conventional van der Waals moiré systems, the constituent layers largely preserve their intralayer bonding networks, while rotational misalignment primarily modulates interlayer tunneling, electrostatic interactions, and structural relaxation [41, 42]. Oxide membranes introduce additional microscopic degrees of freedom because their constituent lattices contain mixed ionic‐covalent bonds and, in transition‐metal perovskites (ABO_3_), three‐dimensionally connected networks of corner‐sharing BO_6_ octahedra. Variations in B─O bond lengths, B─O─B bond angles, octahedral rotation or distortion patterns modify transition‐metal‐oxygen orbital hybridization, crystal‐field splitting, electronic bandwidth, and magnetic anisotropy [72, 73]. At conventional epitaxial oxide interfaces, continuity of the oxygen sublattice can transmit or reconstruct octahedral distortions over several unit cells. In freestanding membranes, however, this bulk octahedral network terminates at the released surfaces. Restacking therefore creates an interface whose local coordination and orbital overlap depend on the identity and relative registry of the exposed atomic planes.

Surface termination constitutes a further degree of freedom that is generally absent from elemental van der Waals layers. AO‐ and BO_2_‐terminated perovskite surfaces expose different atomic species, formal layer charges, coordination environments, and surface dipoles. Consequently, their stacking sequence can modify the local electrostatic boundary condition, interfacial charge distribution, and band alignment. At polar/nonpolar oxide interfaces, termination‐dependent polarity discontinuities are known to drive electronic and/or ionic reconstruction [2, 15]. Interfacial charge redistribution can, in turn, modify metal–oxygen bond geometry, orbital polarization, transport, and magnetic response, illustrating the coupling among charge, lattice, orbital, and spin degrees of freedom [74]. Ferroic order parameters, including polarization, ferroelastic strain, octahedral rotations, magnetization, and antiferromagnetic order, may therefore respond collectively to a spatially varying interfacial environment.

The magnitude, microscopic character, and spatial extent of this coupling are not universal. Freestanding oxide membranes are quasi‐two‐dimensional materials [71], although they are laterally extended and mechanically separable, they retain a finite thickness and a three‐dimensionally connected ionic‐covalent lattice. This distinguishes them from atomically thin van der Waals materials, in which the complete layer thickness may participate directly in the interlayer interaction. In oxide membranes, twist‐induced reconstruction may instead remain confined to the atomic planes adjacent to the interface or propagate farther into the adjoining layers, depending on the microscopic degree of freedom involved. Recent studies illustrate that the registry‐dependent electronic reconstruction in twisted STO is predominantly localized near the interface, whereas strain and polarization reconstruction in twisted BTO can extend farther through the membrane thickness, these material‐specific results are discussed in detail in Section 4 [67, 75]. The effective interaction depth is therefore controlled by the membrane thickness, structural stiffness, dielectric screening, ferroic correlation length, and mechanical and electrostatic boundary conditions.

This spatial hierarchy directly affects experimental observability. Responses confined to one or a few interfacial planes may be diluted in measurements averaged over the full bilayer thickness, whereas structural or ferroic reconstruction extending through a large fraction of the membrane may remain detectable macroscopically. In twisted oxide bilayers, the effective coupling also depends on surface termination, interfacial separation, roughness, contamination, and the degree of atomic‐scale contact; continuous ionic or covalent bonding should therefore not be assumed across the complete interface [65]. Oxide moiré physics is therefore distinguished not simply by stronger interlayer coupling, but by the simultaneous and depth‐dependent modulation of structural coordination, electrostatic potential, orbital hybridization, and coupled ferroic or magnetic order parameters. Within this framework, local atomic registry becomes the fundamental variable connecting twist geometry to interfacial reconstruction.

The registry dependence can first be understood by considering distinct lateral alignments of untwisted or locally commensurate perovskite bilayers. In systems such as SrTiO_3_, these configurations may be represented by atom‐on‐atom AA stacking and laterally displaced AB stacking. The two registries differ in the alignment of the terminating atomic planes and therefore in their local coordination, symmetry, electrostatic environment, and interfacial energy. When a finite twist angle is introduced, the local registry varies continuously across the interface, generating the structural and energetic modulation within the moiré supercell.

The spatial period over which this sequence of local registries repeats is determined jointly by the twist angle and lattice mismatch. For two approximately isotropic lattices, the moiré wavelength is given by

$$\;\lambda_{m}=\frac{\left(1+\delta \right)a}{\sqrt{\delta^{2}+2\left(1+\delta \right)\left(1-\cos\theta \right)}}\;$$

where *a*_1_ and *a*_2_ are the in‐plane lattice parameters of the two layers, δ  = (*a*_2_ − *a*_1_) /*a*_1_is their relative lattice mismatch, and θ is the relative twist angle [76, 77].

For a homobilayer, a_1_ = a_2_  and therefore δ  =  0, the above expression is reduced to

$$\;\lambda_{m}=\frac{a}{2\sin\left(\frac{\theta }{2}\right)}\;$$

demonstrating that the moiré wavelength increases as the twist angle decreases. For small twist angles and weak lattice mismatch, the relation can be approximated as

$$\lambda_{m}\approx \frac{a}{\sqrt{\delta^{2}+\theta^{2}}}$$

with θ expressed in radians. Thus, lattice mismatch can generate a finite moiré period even in the absence of rotation, whereas increasing rotational misalignment generally reduces the characteristic length scale. For anisotropic lattices or layers with inequivalent crystallographic symmetries, the modulation may contain multiple direction‐dependent periodicities and may therefore form a more complex moiré superstructure rather than a single regular scalar period [20, 77].

Within this geometrical framework, the microscopic structure of the moiré supercell can be described in terms of distinct limiting interfacial registries. In the theoretical STO model, the AA and AB stacking configurations can be defined by two similar limiting cases, which are distinguished by the lateral translation of the top layer. In the AA stacking configuration, the top and bottom layers are aligned without any lateral displacement, thus retaining the Ti–O–Ti–O across the interface, similar to the bulk conditions (Figure 3a) [78]. This AA stacking configuration preserves relatively high local symmetry and a uniform electrostatic environment and leads to the continuation of the atomic columns (Ti–Ti and Sr–Sr in STO) across the interface. An analogous atom‐on‐atom alignment can be defined for BaTiO_3_ (BTO) where Ti‐ and Ba‐containing columns coincide across the interface (Figure 3e) [75].

**FIGURE 3:**
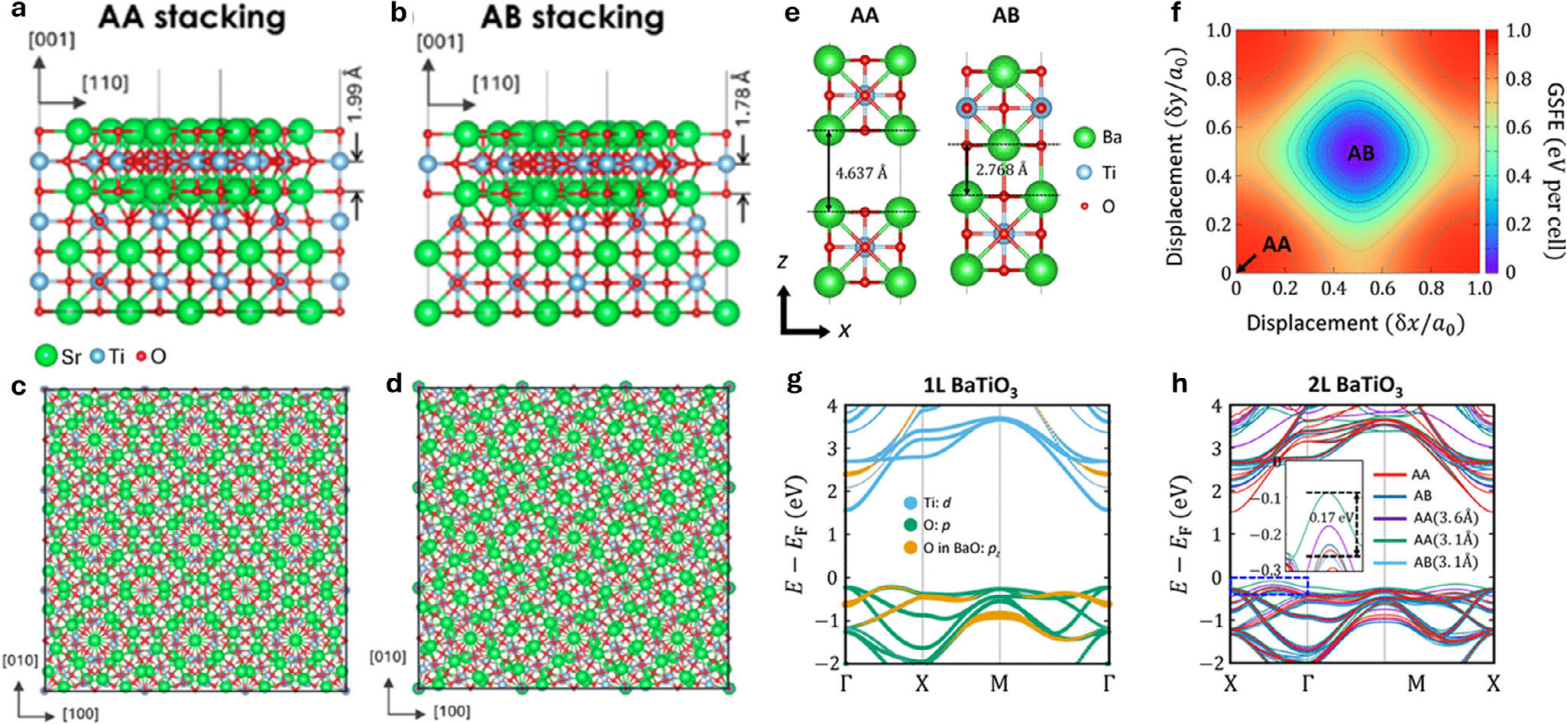
Geometry‐driven interfacial physics in twisted oxide bilayers. (a, b) Atomic structure of AA and AB stacking configurations in perovskite oxides (SrTiO_3_), illustrating the preservation (AA) and disruption (AB) of Ti–O–Ti bonding chains across the interface. Reproduced with permission [78]. Copyright 2025, American Physical Society. (c, d) Real‐space moiré patterns correspond to AA and AB stacking configurations, highlighting distinct spatial arrangements within the moiré supercell arising from differences in local atomic registry. Reproduced with permission [78]. Copyright 2025, American Physical Society. (e) AA and AB stacking configurations in bilayer BaTiO_3_, (f) Generalized stacking fault energy (GSFE) landscape as a function of in‐plane displacement, showing substantial energy variation (∼1 eV per unit cell) between stacking configurations. Reproduced with permission [75]. Copyright 2024, American Association for the Advancement of Science. (g) Electronic structure of monolayer BaTiO_3_. Reproduced with permission [75]. Copyright 2024, American Association for the Advancement of Science. (h) Stacking‐dependent electronic structure of BaTiO_3_ bilayers, illustrating shifts in the valence band maximum (∼0.1–0.2 eV) due to registry‐dependent orbital hybridization and electrostatic effects. Reproduced with permission [75]. Copyright 2024, American Association for the Advancement of Science.

Contrary to AA stacking, AB stacking in STO is obtained by laterally translating the top layer by half a unit cell along the [79] direction of STO (Figure 3b). In the AB configuration, the lateral translation of the top layer disrupts the continuity of the Ti–O–Ti arrangement across the interface, leading to cross‐sublattice alignment (Sr‐Sr) between the two layers. Although the TiO_2_‐SrO in STO or BaO‐TiO_2_ in BTO stacking sequence is preserved in the in‐plane direction in both cases, this misregistry modifies the local coordination environment, results in breaking local inversion symmetry, and generates non‐uniform electrostatic potentials at the interface. From a microscopic perspective, this lateral sliding between the top and bottom layers can be described by generalized stacking fault energy (GSFE), which defines the total energy associated with it as a function of in‐plane displacement vector r. A first‐principles calculation on the BTO bilayer structure reveals that the GSFE landscape exhibits significant energy variation (Figure 3f). The amplitude of this stacking‐dependent energy landscape is substantially larger than that reported for the van der Waals bilayer systems [80, 81], indicating that translation in oxide bilayers is strongly coupled to local coordination, electrostatics, orbital hybridization, and lattice relaxation. Consequently, interlayer sliding cannot generally be treated as a rigid displacement, instead, it is considered as an energetic inequivalent registry that can drive local structural reconstruction and spatially varying interfacial potentials [75].

These structural disorders have a direct influence on the electronic structure through orbital overlaps and local electrostatic variations. As shown in Figure 3g,h, the AA stacking has stronger alignment of oxygen atoms across the interface, leading to a higher valence band maximum (VBM). Whereas for AB stacking, the misalignment of the atoms reduces the overlap and lowers the VBM by ∼0.1–0.2 eV. However, the conduction band remains relatively unaffected. This variation indicates that the stacking configuration generates distinct local energy landscapes for charge carriers. More significantly, this difference in distinct lattice stacking is clearly reflected in real‐space moiré patterns. As demonstrated in Figure 3c,d, AA and AB stacking configurations in STO leads to two distinct spatial arrangements in a 3 × 3 moiré supercell twisted at 22.6°. By introducing a finite twist angle, these stacking‐dependent energy modulations evolve into a continuous spatial modulation of the local potential, resulting in the formation of a moiré supercell. In this regime, AA‐ and AB‐like stacking configurations evolve into a spatially periodic modulation of the local atomic registry across the interface, forming the moiré superlattice. Each region within the moiré unit cell is characterized by distinct local structural relaxations, electrostatic boundary conditions, and electronic band alignments. The resulting moiré potential can be described as a position‐dependent modulation of the interlayer coupling and local electronic Hamiltonian, arising from the interplay between lattice reconstruction, Coulomb interactions, and orbital hybridization. Theoretical models show that a spatially varying registry can generate a position‐dependent modulation of the structural and electronic energy landscape. Depending upon the material composition and boundary conditions, these calculations predict localized electronic states, interfacial dipoles, quasi‐flat bands, and enhanced interaction effects. Some structural and ferroic consequences, including moiré superlattice and vortex‐like polarization textures, have subsequently been observed experimentally. By contrast, the predicted flat bands and associated electronic phases have not yet been directly resolved using experimental measurements.

The energy scale associated with stacking‐dependent interactions in oxide bilayers is significantly larger than in conventional van der Waals systems, the resulting moiré potentials remain robust over a broad range of twist angles. Therefore, twists not only acts as a geometric parameter but also as a powerful tuning knob that continuously modulates the local stacking configuration and, thereby, the interfacial energy landscape. This interplay between twist angle and atomic registry governs the emergence of spatially varying structural, electronic, and ferroic order parameters, enabling phenomena such as charge localization and quasi‐flat electronic bands even at relatively large twist angles (∼10–20° in BaTiO_3_). These insights establish stacking and twist as the fundamental control parameters in oxide moiré systems, providing a theoretical framework for understanding their experimental realization in terms of structural moiré superlattices, polar vortex textures, and emergent magnetic states, which are discussed in the following sections.

## 4 Fundamentals of Moiré Physics in Twisted Complex Oxides

The demand for emergent functionalities in the next generation of electronic devices has significantly increased, and it has pushed research interest from materials discovery to the structural reconfiguration of existing materials. The preceding section will discuss how these periodic modulations originating from twist and stacking can be realized experimentally in terms of structural moiré superlattices, and how these moiré pattern formation results in the formation of polar vortex, correlated electronic states, and flat bands.

### 4.1 Structural Moiré Superlattices in Twisted Oxide Bilayers

As demonstrated in Section 2, experimental realization of moiré superlattices in twisted complex oxide thin films relies on the ability to deterministically stack epitaxial FS membranes with a controlled relative rotation. A key milestone was the demonstration of the formation of moiré patterns in twisted bilayer STO thin films. Atomic‐resolution characterization of twisted FS‐STO thin films obtained from STO/SAO/STO (100) indicates the formation of a sharp and clean interface at a well‐defined relative rotation between two layers, providing an early structural platform for oxide moiré superlattices. A high‐resolution TEM image clearly reveals the moiré pattern in the bilayer region at the twist angle of 22.7° with a moiré periodicity of 14.07 Å (Figure 4a). The moiré pattern in twisted homobilayer STO originates from the twisting of two layers, as there is no lattice mismatch between the top and the bottom layers [20].

**FIGURE 4:**
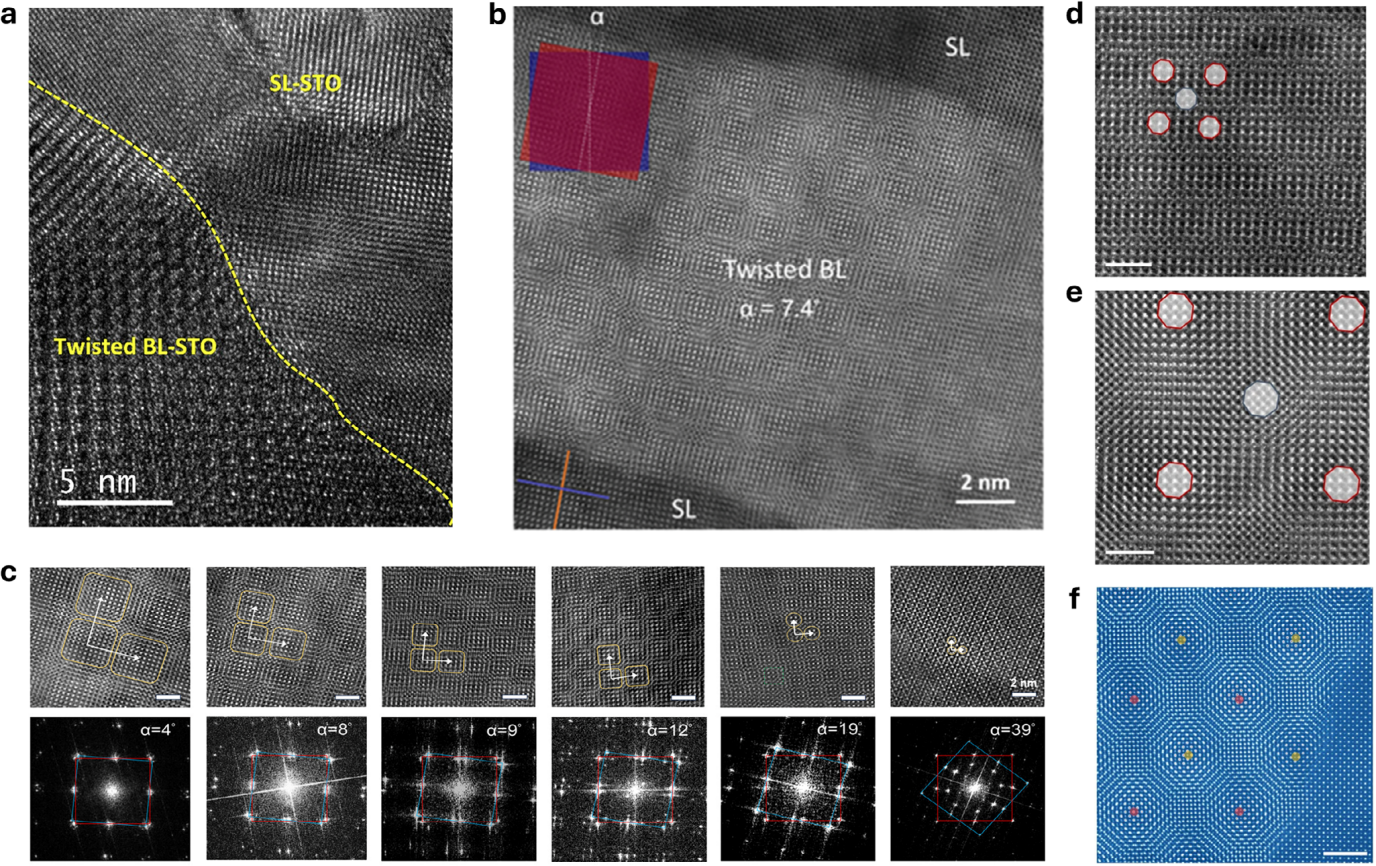
First experimental observation of moiré superlattices in twisted oxide bilayers. (a) HRTEM image of a twisted STO bilayer showing the emergence of a moiré pattern at the interface, representing an early direct observation of twist‐induced structural modulation in oxide systems. Reproduced with permission [20]. Copyright 2022, American Chemical Society. (b) STEM image of twisted La_0.8_Sr_0.2_CoO_3_ (LSCO) membranes displaying moiré contrast in the bilayer region at a finite twist angle (∼7.4°), confirming the formation of rotationally induced superlattices in correlated oxides. Reproduced with permission [82]. Copyright 2025, American Chemical Society. (c) High‐resolution STEM images (top) and corresponding FFTs (bottom) of twisted bilayer LSCO at different twist angles, showing the systematic change in moiré periodicity arising from rotational misalignment. Reproduced with permission [82]. Copyright 2025, American Chemical Society. Plane‐view STEM HAADF image of (d) 10.4° and (e) 3° twisted BaTiO_3_ (BTO) bilayers showing the formation and evolution of the moiré pattern as a function of twist angle. Reproduced with permission [83]. Copyright 2024, Springer Nature (CC BY). (f) Multislice electron ptychography image of a twisted STO bilayer revealing the moiré pattern, with distinct AA and AB stacking regions across the interface. Reproduced with permission [84]. Copyright 2024, Springer Nature (CC BY).

Subsequent work extended this approach to other twisted‐bilayer (TBL) perovskite oxides, establishing the fact that the formation of a moiré superlattice in these twisted bilayers is not limited to STO. In twisted freestanding La_0.8_Sr_0.2_CoO_3_ (LSCO) homobilayers, the twist angle of the sample shown in Figure 4b was determined to be approximately 7.4° from the angular separation between the two sets of fast Fourier transform (FFT) reflections. This value was independently verified using a real‐space moiré‐fringe spacing of 14.91 Å associated with the LSCO (200) lattice planes. The 14.91 Å value therefore represents the fringe spacing used in the twist‐angle analysis and should not be directly compared with the 14.07 Å moiré periodicity reported for STO, because the two values were extracted using different crystallographic and microscopy‐analysis procedures. Within the LSCO series, the spacing between adjacent moiré patterns systematically decreases with increasing twist angle (Figure 4c), supporting their origin in twist‐induced lattice interference rather than strain‐driven reconstruction [82]. Twist‐dependent periodicity established in LSCO was further validated in BTO twisted bilayers, where plane‐view HAADF‐STEM images reveal periodic contrast modulations arising from the interference of two BTO lattices, providing clear evidence of moiré formation at different twist angles (Figure 4d,e). The resulting moiré patterns have distinct highly coincidental regions associated with AA‐ and AB‐like stacking [83]. The change in the moiré wavelength scale with twist angle, further confirms the geometric origin of moiré superlattices in TBL oxides, consistent with the structural trends established in LSCO.

Although these intensity‐based imaging techniques provide significant information about the existence of moiré superlattices in TBL oxides, phase‐sensitive techniques are expected to provide much deeper insight into the structural modulations. The recent developments in multislice electron ptychography have overcome the challenges due to dynamical scattering by treating multiple electron scattering as an inverse problem [85, 86, 87, 88, 89]. This framework allows quantitative reconstruction of the sample's electrostatic potential with sub‐angstrom three‐dimensional (3D) resolution [90]. In STO twisted bilayers, multislice electron ptychography reconstructs the projected electrostatic potential, resulting in direct visualization of the moiré pattern with enhanced sensitivity to subtle structural variations. In the total phase image (Figure 4f), obtained by integrating the reconstructed potential across both layers, the moiré superlattice emerges clearly as a periodic modulation. Distinct coincidence regions can be identified within the supercell: AA‐type regions, where Sr–Sr and TiO–TiO stacking preserves the bulk‐like periodicity, and AB‐type regions, where Sr‐TiO and TiO‐Sr stacking configurations resemble antiphase boundaries. This direct mapping of local stacking configurations highlights the ability of ptychography to resolve registry‐dependent structural variations that are not readily accessible in conventional HAADF‐STEM imaging [84]. Beyond identifying these registry regions, ptychography benefits from improved signal‐to‐noise characteristics and reduced background contributions compared to intensity‐based techniques, particularly in ultrathin oxide membranes. This enhanced phase sensitivity allows visualization of subtle lattice distortions and interfacial modulation associated with the moiré superlattice, providing a more complete description of the reconstructed interface.

These experiments establish the formation of coherent moiré superlattices and spatially varying local stacking configurations in twisted oxide bilayers. Such structural reconstruction provides a microscopic basis from which the electronic and ferroic modulations may emerge, but structural imaging alone does not establish flat‐band formation or correlated electronic phases. The following section therefore distinguishes experimentally reconstructed polarization and charge distributions from electronic states predicted through first‐principles calculations.

### 4.2 Polar Vortex Textures in Twisted Oxide Moiré Superlattices

Polar vortex‐antivortex arrays were first established in conventional epitaxial PbTiO_3_/SrTiO_3_ superlattices, where their stabilization arises from the balance among elastic, electrostatic, and polarization‐gradient energies under substrate‐imposed mechanical and electrical boundary conditions [91]. In such epitaxial systems, the vortex state can be controlled through parameters including lattice mismatch, superlattice periodicity, layer thickness, and electrostatic confinement. Twisted freestanding oxide bilayers extend this concept by introducing the twist angle as an additional geometrical degree of freedom. The continuous variation of atomic registry across the moiré supercell generates spatially periodic lattice rotations, shear strains, and strain gradients, producing a modulated mechanical and electrostatic landscape whose characteristic length scale can be tuned through relative rotation.

Freestanding membrane assembly also expands the available materials‐design space because the constituent layers can be synthesized separately under their preferred growth conditions before being combined. This approach relaxes some of the lattice‐matching, substrate‐clamping, and sequential‐growth constraints of conventional epitaxy and enables interfaces between materials that may be difficult to realize as coherently grown heterostructures. In twisted BTO bilayers, Ti‐displacement maps at twist angles of 10.4° and 3° reveal periodic vortex‐antivortex textures with continuous in‐plane rotation of the polarization around well‐defined cores (Figure 5a,b). Their size and spatial separation follow the moiré modulation, demonstrating that the twist angle provides a direct means of controlling the lateral organization of the polar texture [83]. These polar vortices constitute topologically non‐trivial ferroelectric textures, in contrast to conventional domain structures involving discrete polarization switching. The characteristic size and spacing of the vortex lattice scale with the moiré periodicity, establishing the twist angle as a primary control parameter governing the spatial modulation of polarization [83].

**FIGURE 5:**
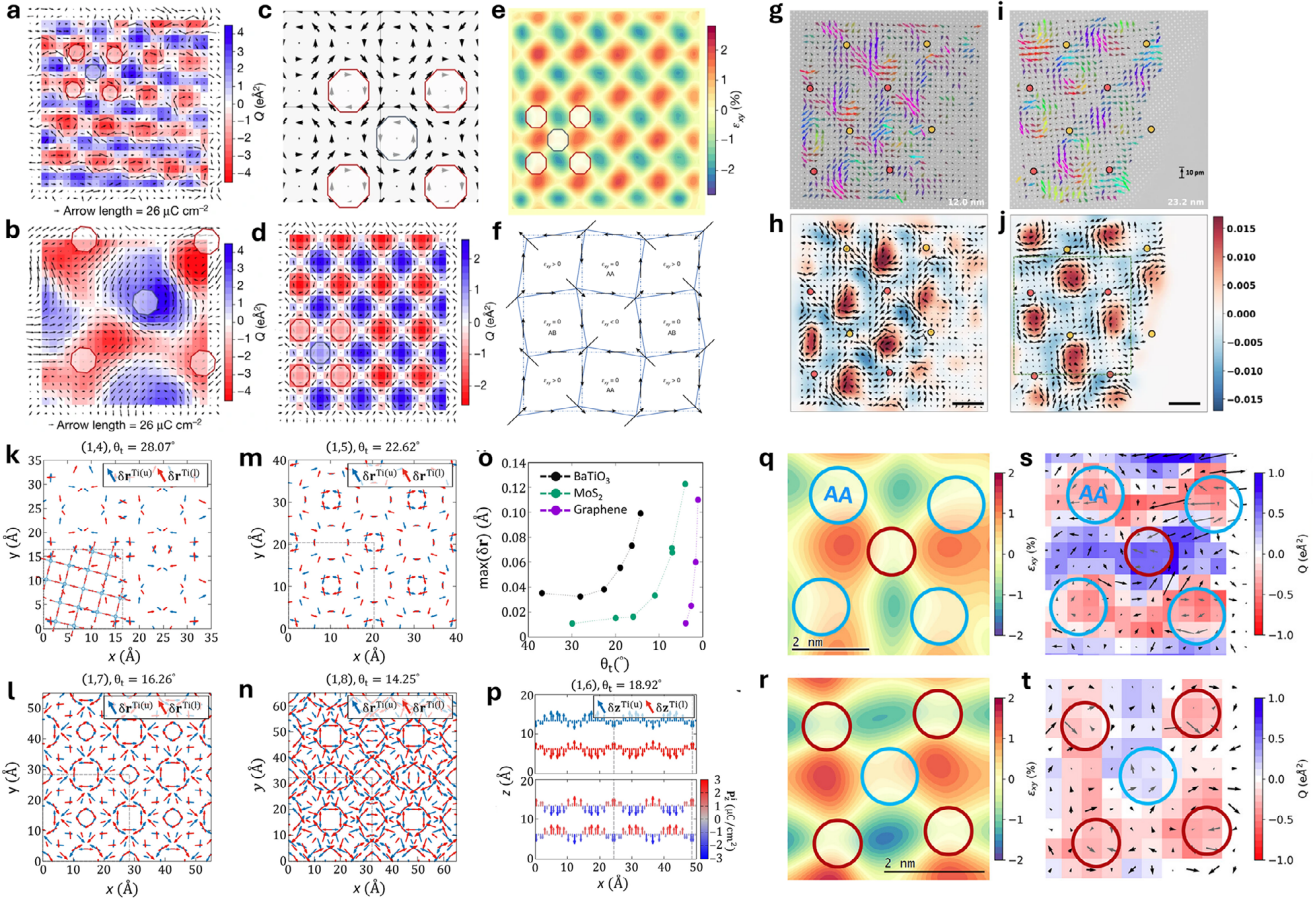
Polar vortex textures in twisted oxide moiré superlattices: from ferroelectric origin to emergent and chiral states. (a, b) Real‐space Ti displacement maps in twisted bilayer BTO at different twist angles (e.g., 10.4° and 3°), revealing the formation of periodic vortex lattices whose size and spacing scale with the moiré periodicity. Reproduced with permission [83]. Copyright 2024, Springer Nature (CC BY). (c–e) First‐principles and continuum modeling of twisted BTO, illustrating the microscopic origin of vortex formation driven by stacking‐dependent energetics and lattice relaxation, as well as the associated polarization rotation and strain distribution. Reproduced with permission [83]. Copyright 2024, Springer Nature (CC BY). (f) Schematic of a twisted BTO moiré supercell showing strain‐gradient‐induced flexoelectric polarization that gives rise to alternating vortex and antivortex textures across AA‐ and AB‐like regions. Reproduced with permission [83]. Copyright 2024, Springer Nature (CC BY). (g–j) Direct experimental observation of vortex‐like polarization textures in twisted STO using multislice electron ptychography, demonstrating emergent polarization and stacking‐dependent structural variations in a nominally paraelectric system. Reproduced with permission [84]. Copyright 2024, Springer Nature (CC BY). (k–p) Theoretical description of vortex structures in twisted BTO, highlighting the role of interlayer coupling in generating chiral polarization textures, out‐of‐plane dipoles, and spatially varying electrostatic potentials. Reproduced with permission [75]. Copyright 2024, American Association for the Advancement of Science. (q–t) Chirality control of polar vortex lattices as a function of twist direction, showing reversal of vortex handedness for opposite twist angles and the emergence of topological ferroic states. Reproduced with permission [92]. Copyright 2026, John Wiley and Sons.

At the microscopic level, density functional theory (DFT) calculations attribute vortex formation to the large stacking‐dependent energy landscape in oxide bilayers, characterized by a substantial generalized stacking fault energy (GSFE). As shown in Figure 5c–e, the spatial variation of stacking configurations across the moiré supercell gives rise to non‐uniform lattice relaxation and associated Ti displacement patterns in 10° twisted BTO bilayers. Each local stacking registry imposes distinct electrostatic boundary conditions and polarization preferences, leading to a spatially frustrated system. The incompatibility between these local minima leads to a frustrated system, which minimizes its free energy through a continuous rotation of polarization rather than abrupt domain formation, thereby stabilizing vortex‐like configurations. In this sense, the vortex lattice emerges as a direct consequence of the interplay between atomic registry, lattice relaxation, and polarization energetics.

A central mechanism underlying this behavior is the presence of strain gradients inherent to the moiré superlattice, which arise from spatially varying lattice reconstruction. As demonstrated in Figure 5f, these strain gradients couple to polarization via the flexoelectric effect, resulting in a vortex‐like modulation of an initially homogeneous ferroelectric polarization state. The observed vortex‐antivortex lattice can thus be interpreted as a flexoelectric ground state, where the polarization continuously adapts to the local strain field to minimize the combined elastic, electrostatic, and polarization‐gradient contributions to the free energy. This framework establishes a direct link between moiré‐induced strain fields and the formation of non‐trivial polarization textures [83]. The continuous rotation of polarization within these moiré‐induced textures places them within the broader family of topological polar structures, which also includes polar skyrmions. However, polar vortices and polar skyrmions are distinguished by the topology of their polarization fields. A polar vortex is characterized primarily by the circulation of polarization around a core, whereas a polar skyrmion involves a continuous three‐dimensional wrapping of the polarization vector and is associated with a nonzero skyrmion number. Polar skyrmion bubbles have previously been demonstrated in PbTiO_3_/SrTiO_3_ heterostructures through control of epitaxial and electrostatic boundary conditions [93]. In contrast, the twisted‐BTO textures were experimentally identified primarily from their in‐plane vortex‐antivortex polarization pattern and were discussed as meron‐like configurations [83]. Establishing a skyrmionic state in twisted oxide bilayers would therefore require reconstruction of the complete three‐dimensional polarization field and evaluation of its topological charge.

The emergence of related vortex‐like polarization textures in nominally paraelectric STO further demonstrates that moiré‐induced polarization reconstruction is not restricted to materials possessing an intrinsic ferroelectric ground state [84]. As shown in Figure 5g–j, multislice electron ptychography enables direct reconstruction of atomic displacements and polarization fields across the interface with sub‐unit‐cell resolution. Despite the absence of spontaneous polarization in bulk STO, the moiré superlattice induces emergent polarization fields through symmetry breaking, interfacial electrostatics, and strain gradients. The measurements further reveal clear differences between AA‐ and AB‐like stacking regions, demonstrating that the local atomic registry governs the spatial variation of polarization. The STO results therefore broaden the range of materials in which moiré‐controlled polar textures may be realized, although they do not imply that vortex formation is universal across all twisted oxide systems.

Theoretical studies further reveal that these vortex structures are inherently three‐dimensional and chiral. First‐principles calculations reveal a vortex‐like polarization texture dominated by in‐plane components, while also indicating the emergence of finite out‐of‐plane dipole moments arising from interlayer coupling and electrostatic effects. This establishes the vortex structure as a three‐dimensional polarization field, despite its predominantly in‐plane character. As shown in Figure 5k–n, Ti‐displacement vector (δ**r** ^Ti^) maps for twisted BTO demonstrate chiral vortex structure for all twist angles. This behavior originates from the large stacking‐dependent energy landscape, characterized by a substantial GSFE, which drives strong lattice relaxation and generates spatially varying electrostatic potentials [75]. Thus, the measured in‐plane displacement for TBL oxides is similar to that in 2D materials at similar twist angle (Figure 5o) [94, 95].

Furthermore, the local atomic displacements (Figure 5p) show non‐ zero out‐of‐plane Ti displacements (δ**z** ^Ti^), defined relative to the surrounding oxygen octahedra. These displacements give rise to a finite local out‐of‐plane polarization P_z_, even at the ultrathin limit where depolarization fields would typically suppress ferroelectricity. As illustrated in Figure 5p, the spatial distribution of P_z_ exhibits a non‐uniform pattern across the moiré supercell, reflecting the underlying stacking‐dependent electrostatic environment. The magnitude of the local dipole moment reaches values on the order of a few µC/cm^2^, comparable to those observed in two‐dimensional ferroelectric materials [96]. Moreover, the calculated P_z_ shows a systematic dependence on the twist angle, indicating that interlayer moiré interactions can stabilize out‐of‐plane polarization in reduced dimensions. These results demonstrate that the vortex structure in twisted oxide bilayers is inherently three‐dimensional, comprising coupled in‐plane rotational polarization and out‐of‐plane dipole modulation. Finally, recent studies demonstrate that the chirality of polar vortex lattices can be tuned by the direction of twist. As shown in Figure 5q–t, reversing the twist direction leads to a corresponding reversal of vortex handedness, reflecting the broken inversion symmetry and chiral nature of interlayer coupling. This provides a direct means of controlling the topological character of polarization textures, enabling the realization of chiral ferroic states that are inaccessible in conventional oxide heterostructures.

Taken together, these results establish moiré modulation as a versatile route for engineering polar‐vortex textures in oxide bilayers. However, strain gradients alone are not sufficient to make vortex formation universal across all twisted oxide systems. Their stabilization depends on the combined effects of stacking‐dependent lattice relaxation, flexoelectric coupling, electrostatic boundary conditions, polarization‐gradient energy, layer thickness, and the dielectric or ferroelectric susceptibility of the constituent materials. The observation of vortex‐like textures in both ferroelectric BTO and nominally paraelectric STO demonstrates that the mechanism is not restricted to intrinsically ferroelectric compounds, while the broader applicability of this behavior remains dependent on the material‐specific energy landscape. Twist should therefore be regarded as an additional and continuously tunable design parameter rather than a universal condition for vortex formation.

### 4.3 Experimentally Resolved Charge Disproportionation and Theoretically Predicted Flat Bands in Twisted Oxide Bilayers

The influence of moiré reconstruction on electronic degrees of freedom has been examined through both spatially resolved experiments and first‐principles calculations. In twisted SrTiO_3_, depth‐sensitive electron microscopy and electron‐energy‐loss spectroscopy provide experimental evidence for registry‐dependent charge disproportionation at the buried interface. By contrast, the associated flat electronic bands in twisted SrTiO_3_, as well as twist‐dependent flat bands and Lieb‐lattice states in twisted BaTiO_3_, have so far been obtained from theoretical calculations. These experimental and theoretical findings are therefore discussed separately below.

#### 4.3.1 Experimentally Resolved Charge Disproportionation in Twisted SrTiO_3_

The electronic structure of TBL STO is governed by the spatial distribution of coincidence‐site‐lattice (CSL) and non‐CSL regions within the moiré supercells as shown in Figure 6a–d [67]. For a twist angle of 10.4°, the Ti‐centered moiré unit cell comprises of four inequivalent local registries (Sr‐, Ti‐, O‐, and non‐CSL environments), each characterized by a distinct interfacial bonding configuration. These four sites can be easily determined in Figure 6e,f. These registries define a spatially varying structural template that directly imprints on the interfacial electronic degrees of freedom. Ti‐L_2,3_ electronic energy loss spectra (EELS) at cation‐centered CSL regions (Sr, Ti) are quite similar to those of bulk STO. However, the spectra in the O‐CSL and non‐CSL region shows significant spectral deviation (Figure 6h) suggesting lattice reconstruction as a function of local electronic structure within the moiré supercell (Figure 6g) [97, 98]. Similarly, from Figure 6i it is evident that electronic reconstruction is confined to the interface as both the *t_2g_* and *e_g_* peaks are well separated in the top and bottom layers. The variation in local coordination environments modifies the Ti‐O hybridization, leading to measurable differences in the Ti electronic configuration across the moiré cell. The absence of a uniform electronic response, despite structural coherence of the interface, establishes direct experimental evidence that the twisted bilayer supports intrinsic charge disproportionation governed by local registry. This registry‐dependent electronic reconstruction is accompanied by the formation of low‐dispersion states at the top of the valence band, giving rise to quasi‐flat bands in the twisted STO bilayer as predicted by DFT (discussed in the next section).

**FIGURE 6:**
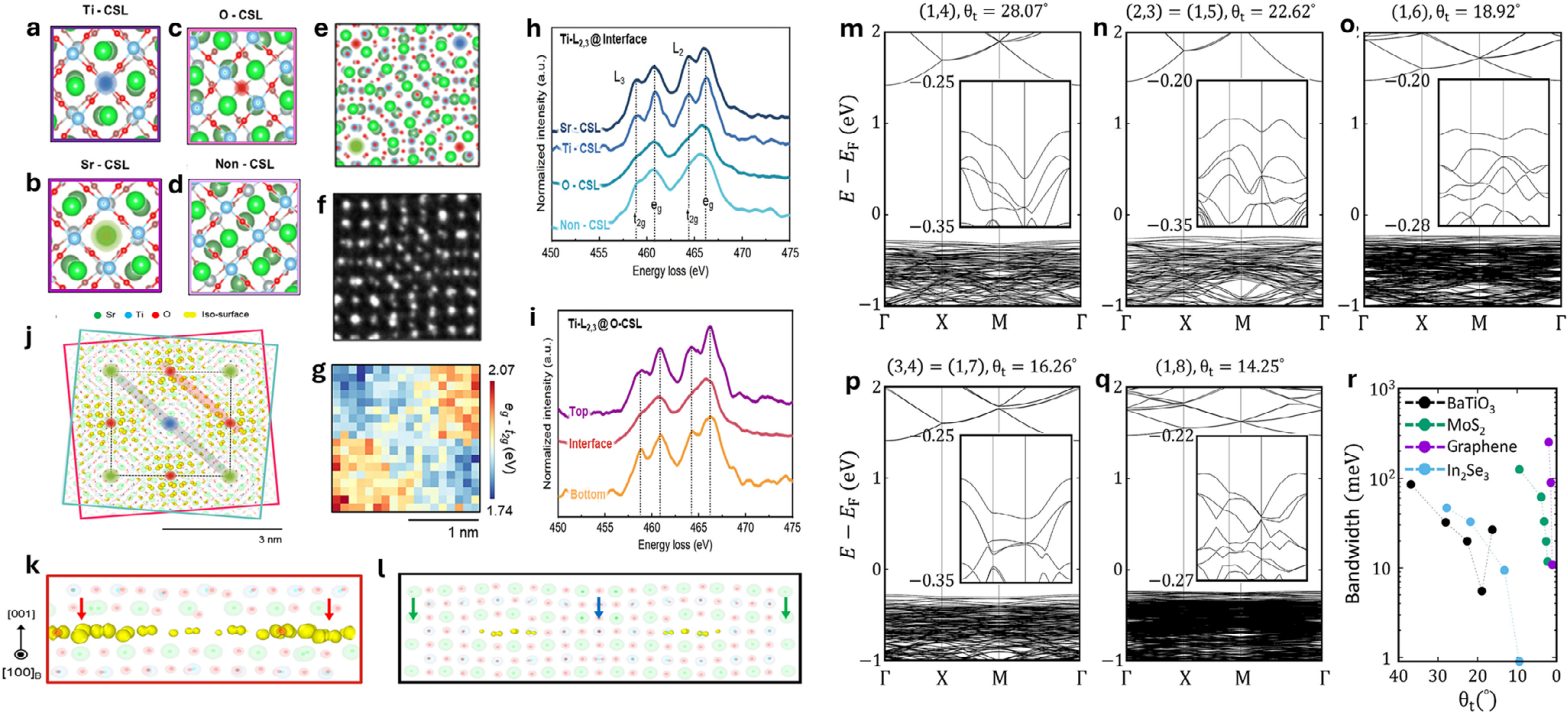
Experimentally resolved moiré‐induced charge modulation and theoretically predicted flat‐band states in twisted oxide bilayers. (a–d) Schematic representations of the distinct coincidence‐site lattice (CSL) environments, Sr‐, Ti‐, O‐, and non‐CSL regions, within the moiré supercell of a 10.4° twisted STO bilayer, illustrating the variation in local coordination geometry at the interface. The interfacial Ti–O bonding configuration differs across these regions, resulting in alternating coordination environments with sixfold symmetry at Sr‐ and Ti‐CSL sites and reduced fivefold coordination at O‐ and non‐CSL sites. Reproduced with permission [67]. Copyright 2025, American Chemical Society. (e) Atomic structure model of the selected moiré region, (f) corresponding atomic‐resolution HAADF‐STEM image, and (g) experimental spatially resolved EELS mapping of the Ti‐L_3_ edge, showing a pronounced reduction in the *e_g_‐t_2g_* energy splitting at O‐CSL regions and a moderate reduction at non‐CSL regions. Reproduced with permission [67]. Copyright 2025, American Chemical Society. (h, i) Experimental EEL spectra acquired from different CSL environments further confirm the modulation of Ti oxidation states, with Sr‐ and Ti‐CSL regions retaining bulk‐like Ti^4^^+^ character, while O‐ and non‐CSL regions exhibit reduced valence states. Reproduced with permission [67]. Copyright 2025, American Chemical Society. (j–l) Theoretical first‐principles calculations showing the real‐space distribution of flat‐band charge density localized at O‐ and non‐CSL regions, with cross‐sectional views demonstrating confinement of these states to the interfacial TiO_2_ layer. Reproduced with permission [67]. Copyright 2025, American Chemical Society. (m–q) Evolution of moiré flat bands based on DFT calculations in twisted bilayer BTO, where the bandwidth decreases with decreasing twist angle, reaching a minimum near the “magic” angle (∼18.9°) and increasing at smaller angles, indicating enhanced electronic localization and the emergence of correlation‐driven flat‐band physics. Reproduced with permission [75]. Copyright 2024, American Association for the Advancement of Science. (r) Comparison of moiré bandwidth obtained for twisted bilayer BTO with 2D materials, including twisted bilayer graphene, MoS_2_, and In_2_Se_3_, highlights that oxide moiré systems can host comparably narrow bands at much larger twist angles, reflecting stronger stacking‐dependent interactions and enhanced electronic localization. Reproduced with permission [75]. Copyright 2024, American Association for the Advancement of Science.

#### 4.3.2 Theoretically Predicted Flat Bands in Twisted SrTiO_3_

Density functional theory (DFT) calculations for a 10.4° twisted STO bilayer reveal the emergence of a flat band at the top of the valence band, characterized by an extremely narrow bandwidth. This flat band originates from a two‐dimensional localized electronic state confined at the interface. The real‐space distribution of the corresponding charge density (Figure 6j–l) shows that the flat‐band states are spatially localized within specific regions of the moiré supercell, namely the O‐ and non‐CSL environments, while being absent in the Sr‐ and Ti‐CSL regions. Cross‐sectional analysis further confirms that this localization is confined to the interfacial TiO_2_ monolayer, indicating that electronic reconstruction is intrinsically interfacial in nature. This spatial selectivity establishes a direct correspondence between local atomic registry and electronic state localization. The origin of this behavior is traced to the reduced coordination at the O‐ and non‐CSL regions, which introduces a dangling‐bond‐like electronic environment at Ti sites. This reduced coordination lowers the Ti valence state and simultaneously generates localized electronic states, leading to a direct correlation between the flat‐band charge density and the Ti oxidation state obtained from Bader charge analysis. In addition, the populated O‐2p orbitals exhibit a zigzag‐like ordering pattern around the CSL regions, indicating that the registry‐dependent structural modulation also drives orbital ordering within the moiré supercell [67].

#### 4.3.3 Theoretically Predicted Flat Bands in Twisted BaTiO_3_

This microscopic picture is consistent with the broader theoretical framework established by recent first‐principles studies of twisted oxide bilayers, which predict that stacking‐dependent variations in local coordination generate spatially modulated electronic potential across the moiré supercell. Within this framework, distinct stacking configurations act as electronically inequivalent sites, producing a periodic variation of the local potential that localizes low‐energy electronic states. The resulting suppression of electronic dispersion can give rise to quasi‐flat bands, conceptually linking the calculated electronic localization to the registry‐dependent reconstruction observed experimentally in twisted STO. A separate first‐principles study of twisted bilayer BaTiO_3_ provides a theoretical framework for understanding how stacking‐dependent structural reconstruction may produce narrow electronic bands in oxide moiré systems. Although this mechanism is conceptually related to the registry‐dependent electronic reconstruction experimentally observed in twisted SrTiO_3_, BaTiO_3_ possesses a distinct ferroelectric ground state, polarization landscape, and interfacial relaxation behavior. The calculated results for twisted BaTiO_3_ should therefore be regarded as an independent theoretical realization of moiré‐induced electronic localization rather than as a direct continuation of the SrTiO_3_ observations.

In the calculated BTO moiré supercells, spatial variations in local stacking configuration generate electronically inequivalent regions and a periodically modulated interfacial potential. This calculated potential landscape localizes selected valence‐band states and reduces their electronic dispersion. As shown in Figure 6m–r, the evolution of the electronic spectrum with twist angle (θ_t_) reveals that the conduction bands remain largely parabolic and weakly perturbed by twist, and the valence band undergoes a pronounced reconstruction. In particular, a detached quasi‐flat band emerges at the top of the valence band over an intermediate range of twist angles (28.07° ≤ θ_t_ ≤ 16.26°), consistent with the formation of localized electronic states within the moiré potential landscape [75]. The degree of band flattening exhibits a non‐monotonic dependence on twist angle. As the twist angle decreases, the bandwidth of the top valence band progressively narrows, reaching a minimum at θ_t_ ≈ 18.92°, before increasing again at smaller angles. This behavior defines a magic‐angle‐like condition, where the flat‐band character is maximized. At this angle, the bandwidth is reduced to ∼5.5 meV, indicating a strong suppression of electronic kinetic energy. Upon further reduction of the twist angle, the separation between the flat band and the remaining valence bands diminishes, eventually leading to band merging and enhanced degeneracy at high‐symmetry points, as observed for θ_t_ = 14.25°. The overall evolution is summarized in Figure 6r, where the bandwidth of the top valence band is plotted as a function of twist angle. The non‐monotonic trend reflects the competition between the moiré length scale and stacking‐dependent electronic reconstruction, which together define the effective potential landscape experienced by the electronic states. The localized states associated with specific registry environments evolve continuously with twist angle, giving rise to a tunable flat‐band regime.

The calculated bandwidths in twisted BaTiO_3_, as well as those predicted for twisted SrTiO_3_, are comparable to the narrow‐band energy scales reported theoretically and experimentally in established moiré platforms such as twisted bilayer graphene [99, 100, 101] and TMDs [102, 103]. Importantly, the oxide calculations predict substantial band flattening at considerably larger twist angles than those generally associated with magic‐angle graphene. This distinction highlights a defining feature of oxide moiré systems where strong interlayer coupling and pronounced stacking‐dependent energetics enable flat‐band formation without requiring ultra‐small twist angles. In addition, unlike van der Waals moiré systems, where the electronic potential varies primarily with the moiré periodicity, twisted oxides exhibit additional intra‐moiré variations arising from the diversity of local coordination environments. This enhanced potential landscape provides a robust mechanism for stabilizing localized states and flat bands even at relatively large twist angles.

The predicted narrow bands reduce the electronic kinetic‐energy scale and may therefore enhance the relative importance of electron‐electron interactions. However, the existence of narrow calculated bands alone does not establish an interaction‐driven correlated phase. Direct experimental measurements of the electronic density of states, band dispersion, carrier transport, and many‐body behavior are still required to verify the predicted flat bands and determine whether they support correlated insulating, magnetic, or other collective electronic states. These theoretical results nevertheless identify twisted BTO as a promising platform in which registry‐dependent reconstruction may generate tunable electronic localization at twist angles substantially larger than those typically investigated in conventional van der Waals moiré systems.

### 4.4 Twist‐Dependent Magnetism and the Emerging Prospect of Oxide Moiré Magnetism

Rotational alignment can modify magnetic behavior in assembled oxide heterostructures even when a moiré magnetic state is not established. In La_0.67_Sr_0.33_MnO_3_(001)/0.7Pb(Mg_1/3_Nb_2/3_)O_3_‐0.3PbTiO_3_(011) (LSMO/PMN‐PT) heterostructures assembled at 0°and 45°, the two configurations exhibited different electric‐field‐induced magnetic anisotropies. The 0°structure developed predominantly uniaxial anisotropy, whereas uniaxial and fourfold components coexisted in the 45°structure. This difference was attributed to the orientation‐dependent transfer of anisotropic piezoelectric strain from PMN‐PT and the resulting magnetoelastic distortion of LSMO [104]. The study therefore demonstrates that rotational alignment provides an additional parameter for controlling magnetoelectric coupling, but it did not investigate or claim a moiré‐periodic structural or magnetic state.

More generally, oxide heterobilayers combining different lattice constants, crystal symmetries, surface orientations, or terminations may develop registry landscapes that are more complex than the comparatively regular moiré patterns of symmetry‐matched homobilayers. Such interfaces may contain competing structural periodicities and spatial variations in strain, bonding, orbital overlap, and magnetic anisotropy that are difficult to describe using a single moiré wavelength. The twist‐dependent response observed in LSMO/PMN‐PT illustrates the sensitivity of oxide magnetism to relative crystallographic orientation, although determining whether analogous direct‐contact heterobilayers develop spatially modulated magnetic interactions will require local structural and magnetic characterization.

A direct oxide‐specific prediction of moiré magnetism has been reported for twisted STO bilayers. First‐principles calculations show that interfacial reconstruction generates high‐density O‐2p states associated with the twist‐modified Ti‐O network. Upon hole doping, exchange splitting of these states produces unconventional *d* ^0^magnetism in otherwise nonmagnetic STO. The calculated spin density is concentrated primarily on interfacial oxygen sites and follows the periodicity of the structural moiré supercell, with its magnitude and onset depending on stacking configuration and carrier filling [78]. This mechanism is distinct from the magnetoelastic response of LSMO/PMN‐PT because the magnetic moments emerge from twist‐induced electronic reconstruction rather than from the modulation of an existing magnetic phase. At present, however, this moiré‐periodic *d* ^0^magnetic state remains a theoretical prediction.

Studies on two‐dimensional van der Waals magnets help clarify magnetic mechanisms that may also be relevant to twisted oxides, but they do not constitute evidence for oxide moiré magnetism. In CrBr_3_ multilayers, differential strain produces a long‐period variation in local stacking and interlayer exchange, leading to regions with different ferromagnetic and antiferromagnetic coupling [105]. Theoretical calculations for twisted CrI_3_ further show that local atomic registry can spatially modulate magnetic exchange and that interfacial Dzyaloshinskii‐Moriya interactions may support non‐collinear or skyrmion‐like spin configurations [106]. Both CrBr_3_ and CrI_3_ are van der Waals magnetic materials rather than oxides. These studies therefore illustrate possible physical mechanisms, whereas registry‐dependent exchange, Dzyaloshinskii‐Moriya interactions, and moiré‐periodic magnetic textures remain unconfirmed in twisted complex‐oxide bilayers.

## 5 Hybrid Oxide‐2D Moiré Heterostructures

Based on the structural and physical background established in twisted bilayer oxides, where controlled twist gives rise to moiré superlattices with spatially modulated lattice, polarization, and electronic degrees of freedom, recent efforts have sought to extend these concepts to hybrid oxide‐two‐dimensional (2D) heterostructures. This extension represents a shift from structurally symmetric systems toward interfaces that combines different lattice symmetries, bonding, and electronic structures. In these hybrid platforms, the interplay between strongly correlated oxide lattices and van der Waals (vdW) 2D materials enables the formation of hybrid moiré superlattices that broaden the scope of conventional moiré physics commonly explored in purely 2D systems. By incorporating the rich ferroic, correlated electronic, spin, orbital, and lattice‐coupled phenomena intrinsic to complex oxides, these oxide‐2D heterostructures provide a highly versatile platform for realizing emergent interfacial states and tunable quantum functionalities with enhanced complexity and diversity. More importantly, such hybridization introduces a fundamentally distinct regime of interfacial physics, where the robust lattice, charge, and polarization degrees of freedom inherent to complex oxides become intimately coupled to the highly tunable electronic structure of vdW materials. In this framework, intrinsic oxide order parameters, including ferroelectric polarization, lattice distortions, octahedral reconstruction, and correlated electronic phases, can serve as spatially modulated moiré potentials that strongly reshape the electronic, magnetic, and optoelectronic responses of the adjacent 2D layer. Consequently, hybrid oxide‐2D moiré heterostructures provide a unique pathway for extending moiré twistronics beyond structurally and electronically symmetric systems, enabling the realization of emergent interfacial phenomena and designer functionalities that are inaccessible in either material platform alone.

Oxide‐TMD heterostructures were explored before the direct observation of hybrid moiré superlattices. Earlier studies demonstrated orientation‐dependent van der Waals epitaxy of monolayer MoS_2_ on crystalline STO surfaces, while freestanding STO membranes were subsequently integrated with two‐dimensional semiconductors as high‐κ dielectric layers [107, 108]. These studies established that crystalline oxides and atomically thin semiconductors can be combined while retaining structurally well‐defined interfaces and functional electronic coupling, although they did not investigate twist‐controlled moiré periodicity or moiré‐induced electronic states.

The realization of a regular oxide‐TMD moiré superlattice depends on the compatibility of the projected in‐plane symmetries of the two constituents, together with their lattice mismatch and relative twist angle. Monolayer WS_2_ possesses a hexagonal lattice, whereas the projected symmetry of cubic STO varies with surface orientation. STO(001) and STO(110) exhibit square and rectangular projected lattices, respectively, while STO(111) presents a pseudo‐hexagonal surface lattice. In the latter case, the effective STO periodicity, $\sqrt{2}a_{\mathrm{STO}}$, is nearly commensurate with $\sqrt{3}a_{WS_{2}}$, enabling the formation of well‐defined and twist‐tunable moiré superlattices at the WS_2_/STO(111) interface [109] as shown in Figure 7c.

**FIGURE 7:**
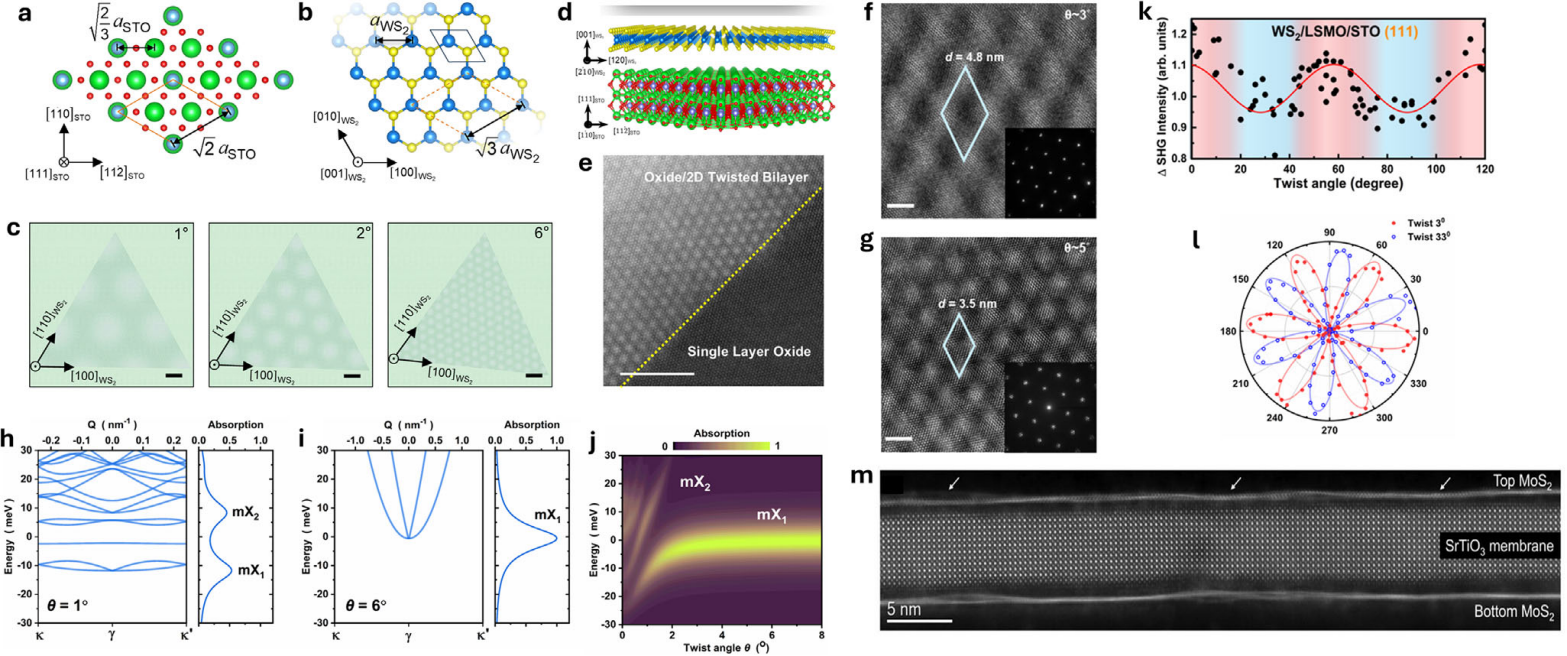
Hybrid moiré superlattices in twisted oxide‐2D heterostructures. Plane‐view schematic of (a) STO and (b) WS_2_ showing hexagonal symmetry and effective primitive cell. Reproduced with permission [109]. Copyright 2026, Springer Nature (CC BY). (c) Simulated moiré patterns in hybrid WS_2_/STO at different twist angles (1°, 2°, and 6°), showing the evolution of the moiré superlattice and a systematic decrease in moiré periodicity with increasing twist angle. Reproduced with permission [109]. Copyright 2026, Springer Nature (CC BY). (d) Cross‐sectional schematic of the WS_2_/STO twisted bilayer illustrating the stacking configuration at the oxide‐TMD interface. Reproduced with permission [109]. Copyright 2026, Springer Nature (CC BY). (e) STEM image of a twisted hybrid WS_2_/STO bilayer, revealing the periodic lattice modulation arising from the rotational alignment between the oxide and 2D layer. The absence of such modulation in the isolated oxide highlights the moiré origin (scale bar = 5 nm). Reproduced with permission [109]. Copyright 2026, Springer Nature (CC BY). HRTEM image of the WS_2_/STO heterostructure at a twist angle of (f) 3° and (g) 5°, with the corresponding SAED pattern (inset) confirming rotational misalignment and moiré periodicity (scale bar = 2 nm). Reproduced with permission [109]. Copyright 2026, Springer Nature (CC BY). (h, i) Continuum‐model calculations of moiré‐exciton miniband dispersions (left) and corresponding optical resonances (right) for twist angles θ = 1° and 6°. Reproduced with permission [109]. Copyright 2026, Springer Nature (CC BY). (j) Color‐coded contour map of moiré‐exciton resonances as a function of twist angle and energy, calculated for a moiré potential depth V_m_ = 50 meV. Reproduced with permission [109]. Copyright 2026, Springer Nature (CC BY). (k) Twist‐angle‐dependent normalized SHG intensity (ΔSHG) of oxide/2D heterostructures, showing a periodic modulation with ∼60° periodicity. Reproduced with permission [109]. Copyright 2026, Springer Nature (CC BY). (l) Linear polarization dependence of SHG intensity for WS_2_ at selected twist angles, preserving the characteristic sixfold symmetry. Reproduced with permission [109]. Copyright 2026, Springer Nature (CC BY). (m) Cross‐sectional HAADF image of MoS_2_/STO/MoS_2_ hybrid 2D‐3D interface. Reproduced with permission [65]. Copyright 2026, John Wiley and Sons.

Against this background, twisted WS_2_/STO(111) heterostructures provided direct experimental evidence of a hybrid oxide‐2D moiré superlattice [109]. Experimentally, the ability to release and transfer ultrathin STO films enables their deterministic stacking with WS_2_, leading to the formation of twisted hybrid heterostructures, as shown in Figure 7d. The formation of a moiré pattern between STO and WS_2_ is evident from the STEM image, confirming the emergence of a periodically modulated lattice, which is absent in the isolated oxide layer shown in Figure 7e. This comparison highlights that the moiré pattern originates specifically from the rotational alignment between the oxide and 2D layer, rather than from intrinsic structural modulation of the individual components. As predicted by simulation, similar to TBL oxides, the moiré periodicity in these hybrid oxide‐2D bilayers also varies systematically with twist angle (Figure 7f,g), reflecting the interplay between lattice mismatch, registry variation, and interfacial coupling. The emergence of twist‐dependent modulation indicates that the moiré superlattice extends beyond a purely structural phenomenon and directly influences the interfacial electronic landscape as shown in Figure 7f,g. Importantly, the significance of this system extends beyond the observation of lattice interference. The differential reflectance (DR) and photoluminescence (PL) measurements reveal the emergence of additional excitonic resonances (mX_1_, mX_2_) on the low‐energy side of the WS_2_ A exciton, which become increasingly pronounced as the twist angle approaches zero and exhibit a systematic redshift with decreasing twist angle, providing clear evidence of moiré exciton minibands. Their temperature robustness, spatial uniformity, and valley polarization confirm their origin as K‐valley neutral excitons confined within a periodic moiré potential, rather than defect‐related states [109].

Continuum modeling reproduced the emergence of multiple bright excitonic states and their twist‐angle‐dependent energy evolution, yielding a moiré‐potential depth of approximately 50 ± 10 meV (Figure 7h–j). First‐principles calculations further reveal that the WS_2_/STO(111) interface exhibits a type‐II band alignment with minimal hybridization at the WS_2_ K valley. Instead, the calculated moiré potential originates predominantly from stacking‐dependent interfacial dipoles associated with the polar STO(111) surface, leading to a bandgap modulation of ∼70 meV across the moiré supercell. The spatial variation of this interfacial dipole modifies the electrostatic potential experienced by WS_2_ and provides an oxide‐specific mechanism for generating the observed exciton minibands. Because polar STO(111) surfaces may undergo electronic, ionic, or stoichiometry‐dependent reconstruction, the magnitude and uniformity of this potential are also expected to depend on surface termination, oxygen stoichiometry, and interface preparation [110].

Beyond excitonic signatures, twist‐dependent interfacial coupling is further reflected in nonlinear optical responses. Second‐harmonic generation (SHG) measurements (Figure 7k,l) exhibit a pronounced twist‐angle‐dependent modulation with a characteristic periodicity of 60°, while preserving the intrinsic sixfold symmetry of WS_2_. This behavior indicates that moiré‐driven variations in interlayer spacing regulate charge transfer at the oxide‐TMD interface, thereby modulating the dielectric response. The WS_2_/STO(111) system therefore represents an important extension of moiré engineering beyond interfaces formed exclusively between van der Waals materials. It demonstrates that a crystalline oxide can act not only as a structural support but also as an active component of a hybrid moiré potential. Its surface symmetry enables the structural superlattice, while its polarity and stacking‐dependent electrostatics govern the resulting electronic modulation. This combination provides access to a moiré potential governed by oxide‐specific degrees of freedom and opens a pathway toward coupling excitonic states in two‐dimensional materials with dielectric, ferroelectric, magnetic, and correlated electronic order parameters available in complex oxides [65].

## 6 Future Opportunities and Challenges in Oxide Twistronics

The rapid progress in twisted bilayer oxides and hybrid oxide‐2D heterostructures has established moiré engineering as a powerful route to tailor structural, ferroic, and electronic properties in complex materials systems. Despite these advances, several key challenges remain in developing this approach into a predictive and scalable platform. In contrast to conventional van der Waals moiré systems, oxide‐based and hybrid moiré architectures involve strong lattice coupling, interfacial polarity, ferroic order parameters, and correlated electronic interactions, substantially increasing both the richness and complexity of the emergent interfacial phenomena. As a result, future progress will require not only improved control over material synthesis and assembly, but also deeper insight into how local structural reconstruction governs the resulting electronic and ferroic responses across multiple length scales.

One central challenge lies in the reliable visualization and interpretation of moiré structures, which depends critically on the choice of characterization technique. Recent studies demonstrate that the same interface can exhibit markedly different moiré contrast when imaged using HAADF‐STEM vs. multislice electron ptychography (MEP), owing to background contributions and thickness sensitivity in intensity‐based imaging (Figure 8a,b). While HAADF‐STEM reflects projected atomic column contrast, MEP reconstructs the projected electrostatic potential with improved signal‐to‐noise and sensitivity to subtle structural modulations. These differences highlight that method‐aware, multimodal characterization is essential for accurately resolving interfacial registry and moiré reconstruction.

**FIGURE 8:**
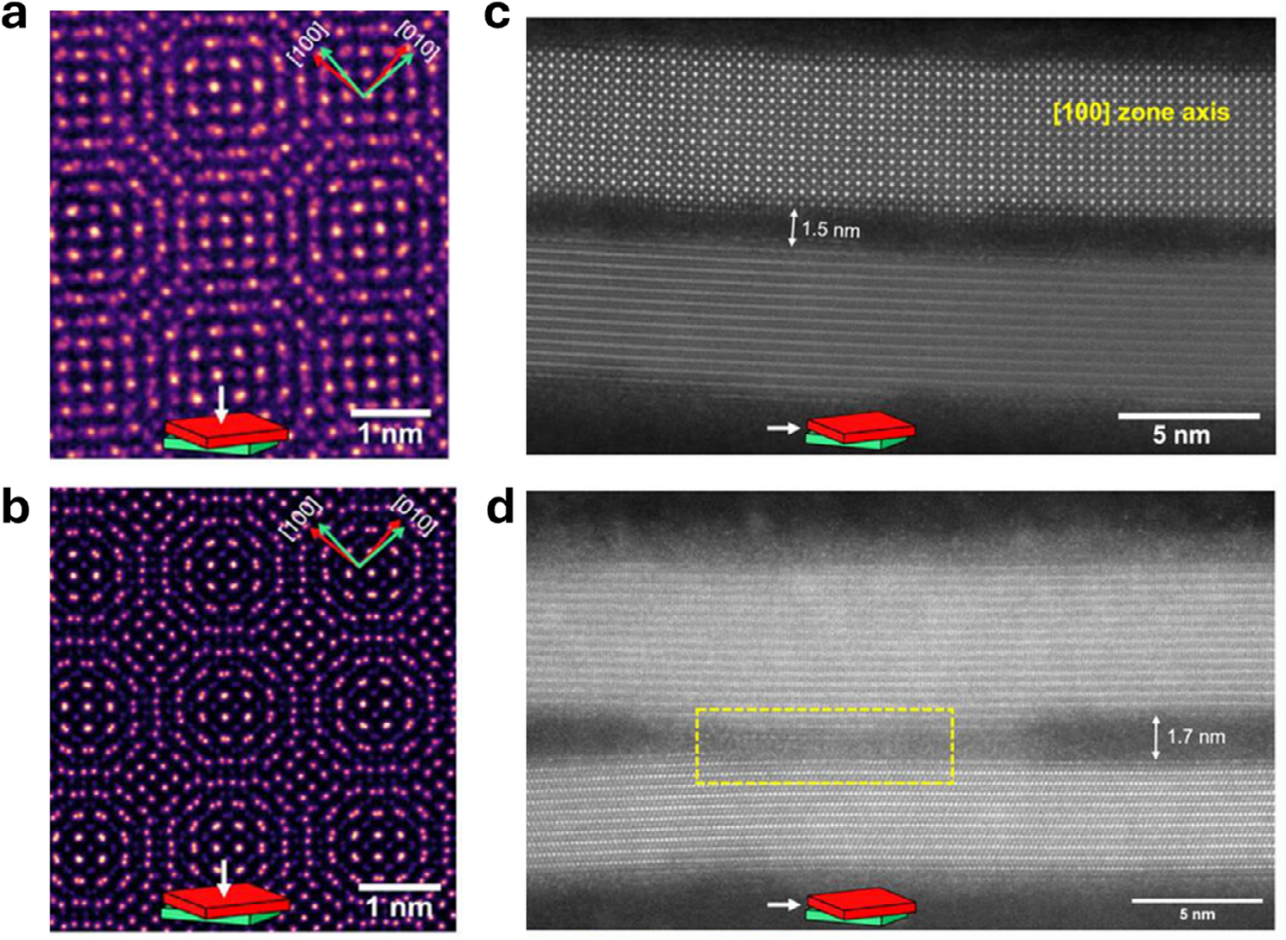
Cross‐sectional and plan‐view imaging of twisted SrTiO_3_ bilayers. (a, b) Plan‐view projections obtained from (a) HAADF imaging and (b) multislice electron ptychography (MEP), showing the moiré superlattice arising from a ∼9° twist. While HAADF contrast is dominated by the top layer, the MEP reconstruction captures contributions from both layers and resolves the oxygen sublattice. Reproduced with permission [65]. Copyright 2026, John Wiley and Sons. (c) Cross‐sectional HAADF image of Sample 1 showing an interfacial gap (∼1.5 nm), indicative of weak interlayer coupling. Reproduced with permission [65]. Copyright 2026, John Wiley and Sons. (d) Cross‐sectional HAADF image of Sample 2 highlighting a localized region of atomic proximity, demonstrating that clean interfaces can be achieved but require controlled surface morphology. Reproduced with permission [65]. Copyright 2026, John Wiley and Sons.

Beyond electron microscopy, scanning‐probe methods provide complementary routes for mapping the functional response generated by a buried moiré interface. A relevant precedent in correlated oxides is provided by LSMO/LAO films, where scattering‐type scanning near‐field optical microscopy resolved the spatial modulation of the electronic response, while magnetic force microscopy revealed the corresponding ferromagnetic texture [44]. Although this moiré pattern originated from the interference of two strain modulations rather than rotational stacking, it demonstrates the value of correlative scanning‐probe measurements for linking an oxide moiré structure to its local electronic and magnetic properties.

For twisted oxide membranes, the appropriate scanning‐probe modality depends on the order parameter of interest. Piezoresponse force microscopy may reveal periodic electromechanical or flexoelectric contrast associated with moiré‐induced strain and polarization reconstruction, while electrostatic force microscopy and Kelvin probe force microscopy can probe surface‐potential modulations generated by buried interfacial dipoles or charge redistribution [79, 111]. Scattering‐type near‐field optical microscopy and nano‐infrared spectroscopy can map spatial variations in optical conductivity, dielectric response, and lattice excitations, whereas scanning microwave impedance microscopy is particularly promising for detecting local conductivity and permittivity at electrically buried oxide interfaces [112, 113]. These techniques have visualized moiré or buried‐interface responses in van der Waals and conventional oxide systems, but their systematic application to twist‐defined oxide bilayers remains largely unexplored.

Because most scanning‐probe signals are not intrinsically confined to the interface, interfacial and non‐interfacial contributions must be separated through correlative measurements and appropriate controls. Functional maps should be acquired over the same region as the structural moiré pattern and compared with adjacent single‐layer regions, crystallographically aligned bilayers, and samples with systematically varied twist angle and membrane thickness. A moiré‐induced response should reproduce the characteristic periodicity and orientation of the structural superlattice and evolve systematically with twist angle, rather than simply following surface topography. Modeling of the tip‐sample transfer function and subtraction of the constituent‐layer response can further quantify the contribution arising from the buried interface.

Magnetic reconstruction requires similarly correlative evidence. For the theoretically predicted hole‐doped STO state [78], electrostatic gating or controlled chemical doping combined with O *K*‐edge and Ti *L*‐edge X‐ray magnetic circular dichroism (XMCD) could test whether the magnetic response originates predominantly from interfacial oxygen states. In intrinsically magnetic oxide bilayers, element‐specific XMCD and X‐ray magnetic linear dichroism (XMLD) could distinguish ferromagnetic, canted, and antiferromagnetic contributions, while resonant elastic X‐ray scattering could search for magnetic satellites at the moiré reciprocal vectors. Polarized neutron reflectometry or resonant magnetic X‐ray reflectometry could further determine whether the magnetic reconstruction is localized near the buried interface. Bulk magnetometry, magnetic hysteresis, and Hall responses may provide supporting evidence, but would not alone establish moiré magnetism without correlation with the structural moiré periodicity and suitable aligned‐bilayer, twist‐angle, thickness, and carrier‐density controls.

Beyond the limitations associated with structural characterization, a central challenge is to distinguish intrinsic moiré reconstruction from spatial variations introduced during membrane assembly. The transfer‐related imperfections discussed in Section 2 can produce local changes in interfacial separation, strain, atomic coordination, and defect density, thereby modifying the effective interlayer coupling across the same nominally twisted interface. As illustrated in Figure 8c,d, transferred oxide membranes may exhibit localized regions of atomic‐scale contact alongside nanometer‐scale interfacial gaps, indicating that intimate coupling is not necessarily maintained uniformly across the bilayer [65]. Variations in membrane roughness, terrace morphology, residual strain, and interfacial cleanliness can therefore modulate the local separation, coordination environment, and effective interlayer interaction within a nominally identical twist geometry. Such spatial heterogeneity may broaden or locally modify the moiré potential and complicate the direct correlation between twist angle, atomic registry, and the resulting electronic, magnetic, or ferroic response. Establishing this correlation will require complementary measurements that combine cross‐sectional imaging with surface topography, local spectroscopy, and chemical analysis before and after transfer. Quantitative evaluation of the contact‐area fraction, interfacial‐gap distribution, roughness, and stoichiometric stability will be essential for comparing fabrication protocols and achieving reproducible device‐scale behavior. These requirements become increasingly important as oxide twistronics progresses from structurally symmetric homobilayers to heterobilayers containing dissimilar lattice parameters, surface terminations, and coupled order parameters.

From a process‐engineering perspective, realizing large‐area, crack‐free, and macroscopically uniform twisted oxide bilayers requires coordinated control over membrane release, mechanical support, rotational alignment, interface formation, and subsequent device fabrication. Current engineering strategies therefore span the complete fabrication sequence, including strain‐compatible sacrificial layers and controlled release chemistry, compliant polymer supports to suppress cracking and wrinkling, calibrated deterministic or tear‐and‐stack assembly to minimize twist‐angle variation, low‐shear lamination and surface preparation to promote conformal contact, and material‐compatible post‐stacking treatments to reduce residual interfacial gaps. During release, controlled etching and drying, together with mechanically compliant support layers, are essential for preserving membrane continuity and limiting strain‐relaxation‐induced damage. During assembly, accurate rotational alignment and low‐shear placement can reduce angular drift, wrinkling, and transfer‐induced deformation, while atomically clean surfaces, controlled termination, and compatible terrace morphology are required to maximize the structurally and electronically active contact area. Any post‐stacking treatment must additionally preserve the oxygen stoichiometry and functional phase of both membranes. Device integration should therefore be preceded by statistical evaluation of the crack‐free overlap yield, wrinkle and bubble densities, spatial variation of the twist angle, contact‐area fraction, interfacial‐gap distribution, functional uniformity, and device‐to‐device reproducibility. Until these parameters can be controlled over macroscopic dimensions, active devices should be patterned only within regions verified as structurally and functionally uniform through correlative mapping.

Such fabrication limitations are not merely technological concerns; they may directly suppress or obscure the emergent phases that motivate oxide twistronics. Phenomena established at conventional oxide interfaces‐including two‐dimensional electron gases, interfacial superconductivity, topological electronic states, and emergent magnetism may, in principle, gain an additional degree of control through twist‐induced modulation of charge, orbital, spin, and lattice interactions in freestanding oxide bilayers. However, cracks, wrinkles, spatially varying twist angles, and nanometer‐scale interfacial gaps disrupt orbital overlaps, exchange pathways, and electrostatic continuity, potentially confining these states to isolated regions or reducing their signatures below the sensitivity of macroscopic measurements. The still‐limited experimental observation of such phases in twisted oxide bilayers should therefore not be attributed solely to an intrinsic limitation of the materials platform, as it may also reflect insufficiently uniform interfacial coupling. Establishing reproducible, strongly coupled, and macroscopically homogeneous interfaces will consequently be essential for determining whether superconducting, topological, magnetic, and twist‐tunable two‐dimensional electronic states can be stabilized and controlled in oxide moiré systems.

A further frontier is the extension of moiré engineering beyond conventional oxide homobilayer architectures. Heterobilayers composed of functionally distinct oxides could combine charge, spin, orbital, lattice, and polarization order parameters within the same moiré‐modulated landscape. Beyond binary oxide‐2D bilayers, a multilayer structure such as MoS_2_/STO/MoS_2_ demonstrates that an ultrathin crystalline STO membrane can be embedded between two TMD layers. Although a twist‐defined moiré superlattice or moiré‐dependent physical response has not yet been demonstrated in this structure, it establishes an important materials platform for future dual‐interface moiré engineering. Independent control of the crystallographic orientation, twist angle, surface termination, and interfacial separation at the upper and lower interfaces could generate coupled or competing periodic potentials across the oxide membrane. Such architectures may provide new routes to manipulate dielectric screening, built‐in electrostatic asymmetry, interfacial charge transfer, exciton confinement, and vertical electronic coupling. A complementary direction for geometry‐controlled oxide design is provided by weave epitaxy. In this approach, epitaxial regions with different crystallographic orientations, ferroic orders, phases, or orbital configurations are laterally connected through atomically sharp in‐plane junctions [24, 114]. Although weave epitaxy does not itself generate an interlayer moiré potential, it extends geometry‐based design to patterned lateral networks and could ultimately be combined with vertically stacked membranes to create integrated lateral‐vertical oxide architectures.

Within this broader architectural landscape, the twist angle provides an additional parameter for controlling structural and functional reconstruction. This opportunity is expected to become particularly important in the small‐angle regime, where lattice relaxation may produce locally preferred stacking domains separated by soliton‐ or dislocation‐like networks. In complex oxides, these boundaries may act as functional elements rather than merely structural imperfections, because their strain fields, altered coordination environments, and defect chemistry can redistribute oxygen vacancies and charge carriers, thereby modifying ionic transport, electronic conduction, and ferroic responses. The accompanying modulation of atomic registry and strain may also reconstruct magnetic exchange, anisotropy, and interfacial Dzyaloshinskii‐Moriya interactions, potentially favoring non‐collinear, skyrmion‐like, or other topological magnetic textures, as discussed in Section 4. Scanning nitrogen‐vacancy magnetometry, which has enabled real‐space imaging of moiré‐modulated magnetic textures in twisted van der Waals magnets, provides a promising approach for testing such behavior in oxide bilayers, particularly when the magnetic period is sufficiently large and the texture generates an uncompensated or canted stray field [106, 115]. Such topological magnetic textures have not yet been experimentally established in twisted oxide systems. Small‐angle oxide twistronics therefore represents a promising direction for jointly engineering stacking domains, dislocation networks, defect‐mediated transport, and magnetic textures through control of twist angle, surface termination, mechanical boundary conditions, and post‐assembly treatment.

From a fundamental perspective, one of the most important open questions concerns the microscopic origin and evolution of moiré potentials across different classes of oxide‐based moiré systems, as well as their direct correlation with emergent functional properties. In twisted bilayer oxides, the moiré landscape is strongly influenced by lattice reconstruction, ferroic order parameters, strain relaxation, and correlated electronic interactions, whereas hybrid oxide‐2D heterostructures introduce additional contributions from interfacial dipoles, dielectric screening, charge transfer, orbital hybridization, and asymmetric bonding environments. These intertwined structural and electronic degrees of freedom substantially enrich the complexity of oxide moiré systems and present new opportunities for exploring emergent interfacial phenomena beyond conventional moiré platforms. While existing theoretical frameworks developed for van der Waals moiré materials provide an important foundation, extending these approaches to incorporate the strong lattice coupling, ferroic interactions, and correlated nature of complex oxides remains a significant challenge.

At the same time, establishing a direct and quantitative relationship between local moiré reconstruction and emergent functionalities remains an outstanding experimental goal. Experiments have established structural moiré superlattices, polar textures, registry‐dependent charge disproportionation, and hybrid oxide‐2D moiré excitonic states. In contrast, direct experimental observations of oxide moiré flat bands, interfacial magnetism, spin‐dependent dynamics, and interaction‐driven correlated phases remain outstanding. Future progress will require magnetic and electronic probes capable of correlating these properties with local atomic registry within individual moiré supercells. Ultimately, the ability to correlate atomic‐scale moiré reconstruction with emergent quantum phenomena will be essential for transforming oxide and hybrid moiré systems into a predictive platform for engineered quantum functionalities, correlated states, and next‐generation oxide nanoelectronics.

## Author Contributions

**Puneet Kaur**: writing – original draft, writing – review and editing, visualization, conceptualization, methodology, validation. **Rahul**: writing – original draft, writing – review and editing, visualization, conceptualization, methodology, validation. **Jan‐Chi Yang**: supervision, project administration, resources, funding acquisition, validation, visualization, writing – review and editing, conceptualization.

## Conflicts of Interest

The authors declare no conflicts of interest.

## Data Availability

No new data were created or analyzed in this study.
